# Epstein-Barr Virus Induced Cytidine Metabolism Roles in Transformed B-Cell Growth and Survival

**DOI:** 10.1128/mBio.01530-21

**Published:** 2021-07-20

**Authors:** Jin Hua Liang, Chong Wang, Stephanie Pei Tung Yiu, Bo Zhao, Rui Guo, Benjamin E. Gewurz

**Affiliations:** a Division of Infectious Disease, Department of Medicine, Brigham and Women’s Hospital, Harvard Medical School, Boston, Massachusetts, USA; b Department of Microbiology, Harvard Medical School, Boston, Massachusetts, USA; c Broad Institute of Harvard and MIT, Cambridge, Massachusetts, USA; d Department of Hematology, First Affiliated Hospital of Nanjing Medical University, Jiangsu Province Hospital, Nanjing, China; e Graduate Program in Virology, Division of Medical Sciences, Harvard Medical School, Boston, Massachusetts, USA; Princeton University

**Keywords:** tumor virus, mononucleosis, chronic active EBV, gammaherpesvirus, nucleotide metabolism, pyrimidine metabolism, primary immunodeficiency, B-cell deficiency, lymphoproliferative disease

## Abstract

Epstein-Barr virus (EBV) is associated with 200,000 cancers annually, including B-cell lymphomas in immunosuppressed hosts. Hypomorphic mutations of the *de novo* pyrimidine synthesis pathway enzyme cytidine 5′ triphosphate synthase 1 (CTPS1) suppress cell-mediated immunity, resulting in fulminant EBV infection and EBV^+^ central nervous system (CNS) lymphomas. Since CTP is a critical precursor for DNA, RNA, and phospholipid synthesis, this observation raises the question of whether the isozyme CTPS2 or cytidine salvage pathways help meet CTP demand in EBV-infected B cells. Here, we found that EBV upregulated CTPS1 and CTPS2 with distinct kinetics in newly infected B cells. While CRISPR CTPS1 knockout caused DNA damage and proliferation defects in lymphoblastoid cell lines (LCLs), which express the EBV latency III program observed in CNS lymphomas, double CTPS1/2 knockout caused stronger phenotypes. EBNA2, MYC, and noncanonical NF-κB positively regulated CTPS1 expression. CTPS1 depletion impaired EBV lytic DNA synthesis, suggesting that latent EBV may drive pathogenesis with CTPS1 deficiency. Cytidine rescued CTPS1/2 deficiency phenotypes in EBV-transformed LCLs and Burkitt B cells, highlighting CTPS1/2 as a potential therapeutic target for EBV-driven lymphoproliferative disorders. Collectively, our results suggest that CTPS1 and CTPS2 have partially redundant roles in EBV-transformed B cells and provide insights into EBV pathogenesis with CTPS1 deficiency.

## INTRODUCTION

Epstein-Barr virus (EBV) causes infectious mononucleosis and persistently infects 95% of adults worldwide. For most individuals, lifelong EBV colonization of the memory B-cell compartment is asymptomatic. Nonetheless, EBV is associated with 200,000 human cancers per year, including Burkitt lymphoma, posttransplant lymphoproliferative diseases, Hodgkin lymphoma, and gastric and nasopharyngeal carcinoma (1, 2). Endemic Burkitt lymphoma caused by EBV remains the most common childhood cancer in sub-Saharan Africa (3–5).

EBV is also associated with a growing number of rare congenital immunodeficiency syndromes, in which impairment of cell-mediated immune responses disrupts the host/virus balance (6, 7). Inborn errors of immunity associated with severe EBV disease include *SH2D1A* and *XIAP* mutations, which cause the X-linked lymphoproliferative diseases 1 and 2 syndromes, respectively (8). Severe EBV infection is also associated with deficiency of the MAGT1 transporter, GATA2, the interleukin-2 (IL-2)-inducible T-cell kinase, the RAS guanyl-releasing protein 1 RASGRP1, CD27, CD70, and 4-1BB (6, 7, 9–13). Chronic active EBV and EBV^+^ lymphomas occur with gain-of-function phosphatidylinositol 3-kinase catalytic subunit mutations that cause T-cell senescence (14).

Autosomal recessive mutations of the cytidine 5′ triphosphate synthetase 1 *CTPS1* gene cause a primary immunodeficiency syndrome characterized by impaired T-cell proliferation, low numbers of invariant and mucosa-associated T cells and NK cells, and susceptibility to encapsulated bacterial and chronic viral infections, particularly by EBV and the related varicella-zoster virus (15, 16). Most CTPS1-deficient patients experienced EBV disease, including severe infectious mononucleosis, chronic EBV viremia, and EBV-associated primary CNS lymphomas (16). Extrahematopoietic manifestations of CTPS1 deficiency have not been reported, and hematopoietic stem cell transplantation is curative (17).

**FIG 1:**
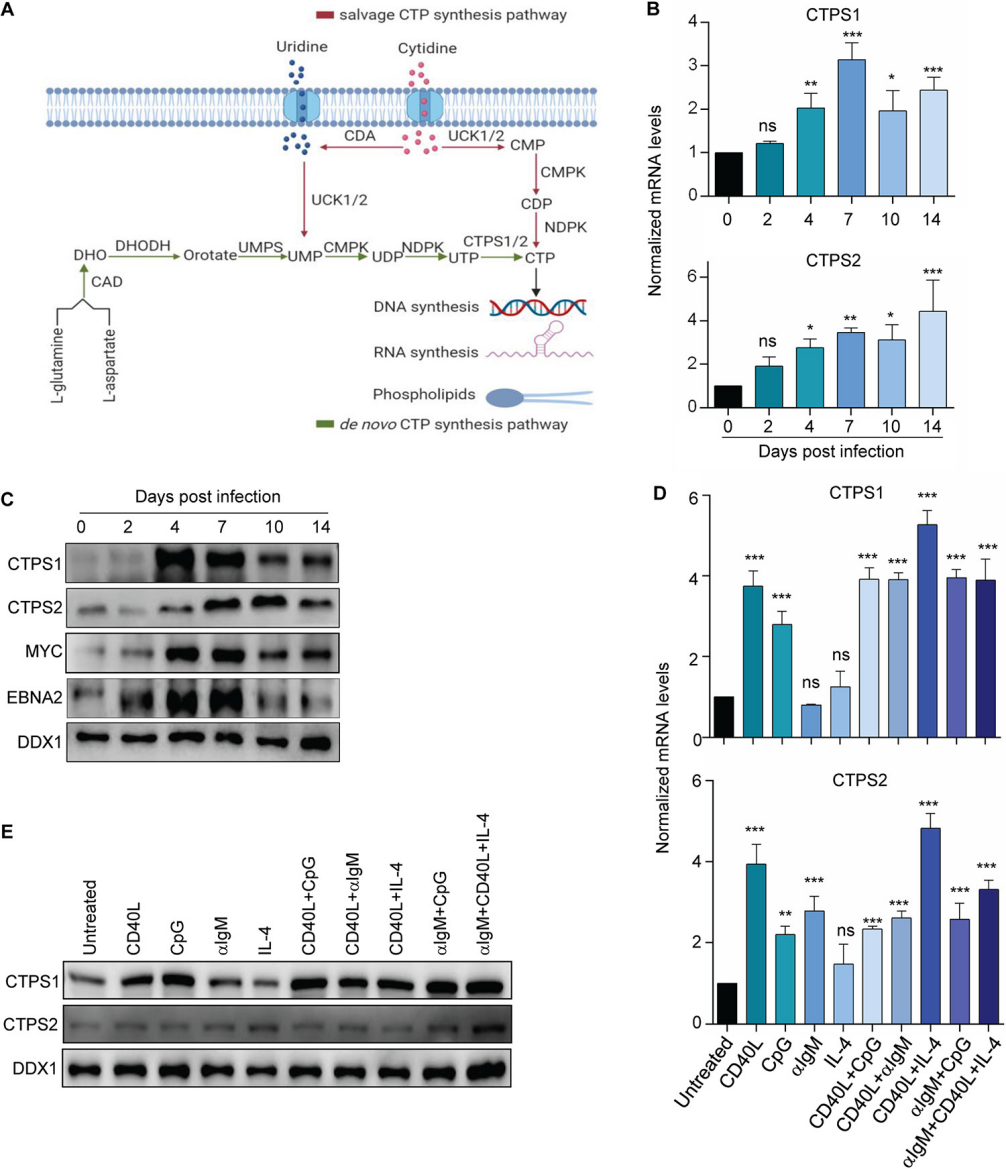
Upregulation of *de novo* CTP synthesis enzymes CTPS1 and CTPS2 in primary human B cells stimulated by EBV or B-cell agonists. (A) Schematic diagram of *de novo* and salvage CTP synthesis pathways. The *de novo* pathway synthesizes CTP from l-glutamine and l-aspartate or uridine substrates, whereas the salvage pathway uses cytidine for CTP synthesis. (B) Quantitative PCR (qPCR) analysis of CTPS1 and CTPS2 mRNA levels in primary human CD19^+^ peripheral blood B cells infected by EBV B95.8 strain for the indicated days. (C) Immunoblot analysis of CTPS1, CTPS2, MYC, EBNA2, and load control DDX1 levels in whole-cell lysate (WCL) from primary human CD19^+^ peripheral blood B cells infected by the EBV B95.8 strain for the indicated days. DDX1 was used for load control since GAPDH and tubulin levels change in primary B cells upon EBV infection, but DDX1 levels remain relatively constant over the first month of EBV infection (28, 29). (D) qPCR analysis CTPS1 and CTPS2 mRNA levels in primary human CD19^+^ B cells stimulated for 24 h by Mega-CD40 ligand (CD40L, 50 ng/ml), the Toll receptor 9 agonist CpG (1 μM), αIgM cross-linking (1 μg/ml), interleukin-4 (IL-4, 20 ng/ml), or combinations thereof, as indicated. (E) Immunoblot analysis of CTPS1, CTPS2, and DDX1 abundances in WCL of primary human CD19^+^ B cells stimulated for 24 h by CD40 ligand Meda-CD40L (50 ng/ml), CpG (1 μM), αIgM (1 μg/ml), IL-4 (20 ng/ml), or combinations thereof, as indicated. For panels B and D, mean and SEM values from *n* = 3 replicates are shown. *, *P < *0.05; **, *P < *0.01; ***, *P < *0.001; ****, *P < *0.0001; ns, nonsignificant using one-way ANOVA with multiple comparisons. For panels C and E, representative blots from *n* = 3 replicates are shown.

The nucleotide cytidine is a key precursor for DNA, RNA and phospholipid biosynthesis, as well as protein sialyation. CTP is maintained at the lowest concentration of cellular nucleotide pools, and its synthesis is tightly regulated (18, 19). CTP is produced through *de novo* or salvage pathways. The isozymes CTPS1 and CTPS2 catalyze the ATP-dependent transfer of nitrogen form glutamine to UTP, thereby producing CTP and glutamate. CTPS1 and -2 share 74% amino acid sequence identity (20, 21). CTP can also be synthesized by salvage pathways, where uridine or cytidine is imported and phosphorylated by the enzyme uridine-cytidine kinase 1 (UCK1) or UCK2. CMP is subsequently converted to CTP by the kinases CMPK and NDPK (Fig. 1A). CTP synthases are the rate-limiting enzymes in *de novo* CTP synthesis (22), and CTPS1 is rapidly and highly upregulated in primary human T cells upon T-cell receptor stimulation (23).

All patients described thus far with CTPS1 deficiency harbor a homozygous missense G→C mutation at base 1692 of the *CTPS1* gene (rs145092287) that alters an intronic splice acceptor site. While the mutation does not alter CTPS1 catalytic activity, it causes 80 to 90% loss of CTPS1 protein abundance (16). The high prevalence of B-cell driven EBV-associated diseases in people with CTPS1 deficiency raises the question of how EBV-driven lymphomas arise at high frequency, despite pronounced defects in lymphocyte metabolism. Patients with CTPS1 deficiency have normal total T- and B-cell numbers but a reduced frequency of memory B cells, the site of long-term EBV persistence (16). Whether CTPS2 may have more important roles in EBV-infected B cells than in T and NK cells and how CTPS1 deficiency alters the latent versus lytic stages of the EBV life cycle remain unknown.

B cells from individuals with CTPS1 deficiency exhibit defects in proliferation (16, 23). Furthermore, the *de novo* pyrimidine synthesis enzyme DHODH inhibitor leflunomide impairs growth of EBV-driven lymphomas in murine models (24). Nonetheless, continuously growing EBV-transformed lymphoblastoid cell lines (LCLs) can be established from B cells of patients with CTPS1 deficiency (16, 23). LCLs express the EBV latency III program also observed in primary CNS lymphomas, comprised by six Epstein-Barr nuclear antigens (EBNA) and two latent membrane proteins (LMPs) that mimic signaling by the B-cell receptor and CD40 (25, 26).

To gain insights into EBV pathogenesis in the setting of CTPS1 deficiency, we characterized roles of CTPS1, CTPS2, and cytidine salvage pathways in support of EBV-transformed B-cell growth and lytic replication.

## RESULTS

### EBV upregulates CTPS1 in newly infected B cells.

To gain insights into EBV effects on CTPS1 and CTPS2 expression, we profiled primary human B cells at rest and at five time points postinfection. Whereas low levels of CTPS1 or CTPS2 were detected in resting peripheral blood CD19^+^ B cells, EBV markedly upregulated both CTPS1 and CTPS2 mRNAs. CTPS1 mRNA reached maximum levels between days 4 and 7, the period where transforming cells undergo Burkitt-like hyperproliferation (27, 28). In contrast, CTPS2 was progressively upregulated as newly infected cells converted to lymphoblastoid physiology between days 7 to 14 postinfection (Fig. 1B). Similar effects were evident on the protein level (Fig. 1C), where CTPS1 exhibited comparable expression patterns to Epstein-Barr nuclear antigen 2 (EBNA2) and MYC, each of which are important metabolism regulators. Using our recently published transcriptome sequencing (RNA-seq) and proteomic maps of EBV-driven B-cell growth transformation (28, 29), EBV was also found to upregulate nearly all components of the pyrimidine *de novo* and salvage pathways at similar time points (see Fig. S1A to F).

10.1128/mBio.01530-21.2 FIG S1 EBV-induced *de novo* pyrimidine synthesis pathway expression. (A to F) CAD (A), DHODH (B), UMPS (C), CMPK1 and CMPK2 (D), CTPS1 and CTPS2 (E), NME1 and NME2 encoded NDPK (F) relative protein abundances (top) and mRNA abundances (bottom) over the first 28 days of peripheral blood CD19^+^ B-cell infection by EBV B95.8 at a multiplicity of infection of 0.1. Mean plus the SEM values from *n* = 4 tandem-mass-tag-based proteomic and *n* = 3 RNA-seq replicates were used for these analyses and were obtained from recently published datasets (28, 29). *, *P < *0.05; **, *P < *0.01; ***, *P < *0.001; ****, *P < *0.0001; ns, nonsignificant, using one-way ANOVA with multiple comparisons. Download FIG S1, TIF file, 2.6 MB. Copyright © 2021 Liang et al. 2021 Liang et al. https://creativecommons.org/licenses/by/4.0/ This content is distributed under the terms of the Creative Commons Attribution 4.0 International license.

CTPS1 and 2 are upregulated in primary B cells activated by combinatorial mitogenic stimuli, including by CD40-ligand (CD40L) plus IL-4, but responses to individual stimuli have not been studied (23). To cross-compare immune receptor and EBV effects on CTPS1 and CTPS2 expression, CD19^+^ primary B cells were stimulated for 24 h with CD40L, the Toll-like receptor 9 agonist CpG, αIgM, the cytokine IL-4, or combinations thereof (Fig. 1D and E). Interestingly, CD40L or CpG alone were sufficient to upregulate CTPS1 mRNA and protein to near maximal levels, whereas CTPS2 was significantly induced on the mRNA but not protein level in response to most B-cell agonists (Fig. 1D and E). These results highlight dynamic CTPS1 and CTPS2 regulation that varies over phases of EBV-mediated B-cell transformation and in response to particular receptor agonists.

### CTPS1 is important for EBV-infected B-cell outgrowth.

**FIG 2:**
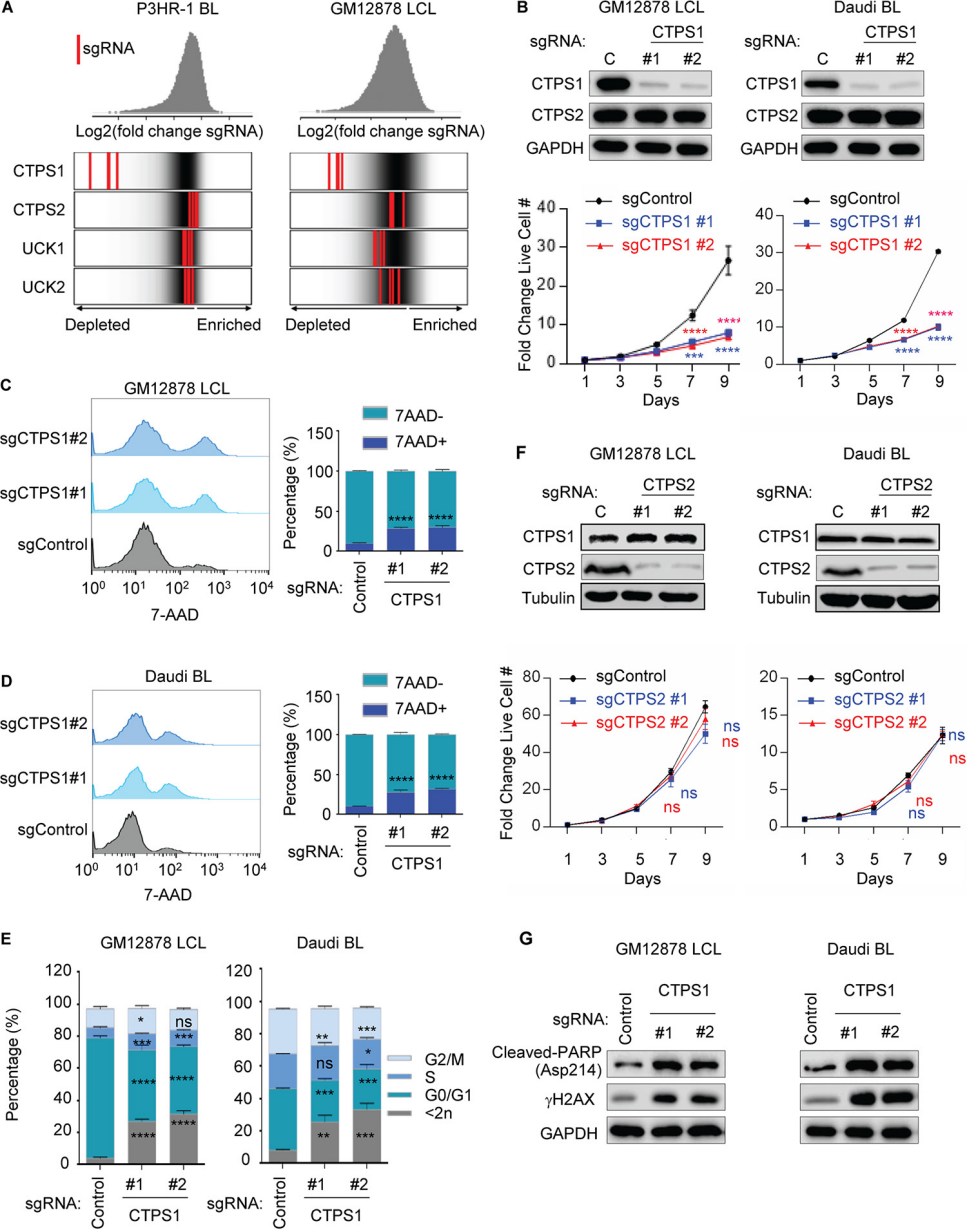
CTPS1 roles in EBV transformed B-cell growth and survival. (A) Distribution of Avana human genome-wide CRISPR screen sgRNA log_2_-fold change values at day 21 versus input in Cas9^+^ P3HR-1 Burkitt lymphoma (BL) left or GM12878 LCL (right). Values for CTPS1, CTPS2, UCK1, or UCK2 targeting sgRNAs (red lines) are overlaid on gray gradients depicting all Avana sgRNA library values (30). Average day 21 versus input values from four screen biological replicates are shown. (B) Immunoblot analysis (top) and growth curve analysis (bottom) of Cas9^+^ GM12878 LCLs or Daudi BL expressing nontargeting control (lanes C) or independent sgRNAs against *CTPS1*. WCL were obtained at day 7 after sgRNA expression. Growth curves were begun 96 h after sgRNA expression and 48 h after puromycin selection. (C) Flow cytometry (FACS) analysis of vital dye 7-AAD uptake in GM12878 LCL at day 7 after expression of control or sgRNAs targeting *CTPS1*. Shown at right are median plus SEM values from *n* = 3 replicates. (D) FACS analysis of 7-AAD uptake in Daudi BL at day 7 after expression of control or *CTPS1* targeting sgRNAs. Shown at right are median plus the SEM values from *n* = 3 replicates. (E) Propidium iodide cell cycle analysis of GM12878 LCL (left) or Daudi BL (right) expressing control or CTPS1 sgRNAs at day 7. (F) Immunoblot (top) and growth curve analysis (bottom) of GM12878 or Daudi expressing control or independent sgRNAs against CTPS2. WCL were obtained at day 5 after sgRNA expression. Growth curves were begun at 96 h after sgRNA expression and 48 h after puromycin selection. (G) Immunoblot analysis of WCL from GM12878 or Daudi, 7 days after expression of control or CTPS1 sgRNAs. For panels B, C, D, E, and F, mean and SEM values from *n* = 3 replicates are shown. *, *P < *0.05; **, *P < *0.01; ***, *P < *0.001; ****, *P < *0.0001; ns, nonsignificant using one-way ANOVA with multiple comparisons. For panels B, F, and G, representative blots or *n* = 3 replicates are shown.

Since LCLs from CTPS1-deficient patients retain 10 to 15% CTPS1 expression, this residual CTPS1 activity may be necessary for EBV-transformed B-cell growth or survival. Alternatively, CTPS2 redundancy may support these functions. To investigate these possibilities, we first reanalyzed data from our recent human genome-wide CRISPR/Cas9 growth and survival screen (30). Strong selection against all four single-guide RNAs (sgRNAs) targeting the *CTPS1* gene was evident at screen day 21 of the LCL GM12878 and the EBV^+^ Burkitt cell line P3HR-1. In contrast, sgRNAs against *CTPS2* or the genes encoding pyrimidine salvage pathway enzymes UCK1 or UCK2 were not strongly selected against, suggesting that functional knockout of their targets were tolerated by these EBV-transformed B-cell lines (Fig. 2A). Next, CRISPR mutagenesis was performed with *CTPS1* targeting sgRNAs to characterize acute CTPS1 loss-of-function effects on Burkitt and LCL proliferation (Fig. 2B). Live cell numbers were measured at five time points following expression of control versus independent *CTPS1* targeting sgRNAs and puromycin selection. CTPS1 depletion significantly impaired growth of the EBV^+^ Burkitt cell line Daudi, which has latency I expression, and of the LCL GM12878 (Fig. 2B). CTPS1 editing also diminished proliferation of GM11830 LCLs and Mutu I Burkitt cells (see Fig. S2A and B). A low level of proliferation was nonetheless observed in CTPS1-depleted cells, suggesting alternative routes of CTP synthesis.

10.1128/mBio.01530-21.3 FIG S2 CTPS1 roles in EBV-transformed B-cell growth and survival. (A and B) Immunoblot analysis (top) and growth curve analysis (bottom) of GM11830 LCLs (A) and Mutu I BLs (B) expressing control or independent sgRNAs against CTPS1. WCL were obtained at day 7 after sgRNA expression. (C) FACS analysis of 7-AAD uptake in GM11830 LCL (left) or Mutu I BL (right) expressing control or CTPS1 sgRNAs for 7 days. (D) Median plus the SEM values from *n* = 3 replicates, as in panel C. (E) Propidium iodide cell cycle analysis of GM12878 LCL, GM11830, Daudi BL, or Mutu I BL expressing control or CTPS1 sgRNAs at day 9 after sgRNA expression. (F) Median plus the SEM values from *n* = 3 replicates, as in panel E. The percentages of cells in G_2_/M, S, G_0_/G_1_, or with <2*n* DNA content indicative of cell death are shown. (G) Immunoblot analysis of WCL from GM11830 (left) or Mutu I cells (right) expressing control or CTPS1 sgRNAs for 7 days. Blots in panels A and G are representative of *n* = 3 experiments. Mean plus SEM values are shown in panels A, D, and F from *n* = 3 replicates. *, *P < *0.05; **, *P < *0.01; ***, *P < *0.001; ****, *P < *0.0001; ns, nonsignificant using one-way ANOVA with multiple comparisons. Download FIG S2, TIF file, 1.5 MB. Copyright © 2021 Liang et al. 2021 Liang et al. https://creativecommons.org/licenses/by/4.0/ This content is distributed under the terms of the Creative Commons Attribution 4.0 International license.

To examine effects of CTPS1 depletion on B-cell survival, 7-Aminoactinomycin D (7-AAD) vital dye exclusion assays were performed on LCLs and Burkitt cells 5 days after sgRNA expression and puromycin selection, the time point at which growth curves of control and CTPS1 depleted cells diverged. Loss of CTPS1 significantly increased 7-AAD uptake in GM12878 and Daudi cells (Fig. 2C and D) and in a second LCL and Burkitt pair (see Fig. S2C and D), indicating induction of cell death. Cell cycle analysis demonstrated that CTPS1 editing also impaired cell growth, with arrest most pronounced at the G_1_/S stage (Fig. 2E; see also Fig. S2E and F). Depletion of CTPS1 induced phosphorylation of H2AX at Ser 139 (γ-H2AX), indicative of DNA damage, perhaps results from imbalance in CTP nucleotide pools. PARP cleavage was also evident, indicating apoptosis induction (Fig. 2G; see also Fig. S2G).

### Compensatory CTPS2 role in EBV-transformed B-cell outgrowth.

EBV^+^ CNS lymphomas are often observed in patients with CTPS1 deficiency, which are estimated to have 10 to 20% residual CTPS1 activity (16). However, the CRISPR Cancer Dependency Map (DepMap) found that targeting of CTPS2 only mildly affects proliferation of a broad range of cancer cells, including B-cell lymphomas (see Fig. S3A). These observations raise the question of the extent to which CTPS2 can be recruited to support *de novo* salvage CTP synthesis in EBV-transformed B cells in the context of hypomorphic CTPS1 activity.

10.1128/mBio.01530-21.4 FIG S3 Analysis of CTPS2 roles in EBV-transformed B-cell growth. (A) DepMap CRISPR screen dependency scores (56) for sgRNAs targeting CTPS1 (left) and CTPS2 (right) across cell lines from the indicated cancer cells of origin. Each oval represents the DepMap screen value for a cell line from the tissue indicated at right. Negative values indicate selection against the sgRNAs target the gene over a 21-day growth and survival screen. Values <–1 (red vertical line) indicates cell line dependency on the targeted gene for growth or survival. (B and C) Immunoblot analysis (top) and growth curve analysis (bottom) of GM11830 LCL (B) or Mutu I BL (C) expressing control or independent CTPS2 sgRNAs for 7 days. (D) FACS analysis of 7-AAD uptake in GM12878 (left) or Daudi cells (right) expressing control or CTPS2 sgRNAs for 7 days. (E) Median plus SEM values from *n* = 3 replicates, as in panel D, of 7-AAD uptake FACS analysis in GM12878, GM11830, Daudi, or Mutu I cells. (F) Mean plus SEM values of FACS propidium iodide cell cycle analysis of GM12878 (left) or Daudi (right) expressing control, CTPS2, CTPS1, or CTPS1/2 sgRNAs for 7 days. Significantly differences from sgControl values are indicated. Blots in panels B and C are representative of *n* = 3 experiments. Mean plus SEM values are shown in panels B to F are from *n* = 3 replicates. *, *P < *0.05; **, *P < *0.01; ***, *P < *0.001; ****, *P < *0.0001; ns, nonsignificant using one-way ANOVA with multiple comparisons. Download FIG S3, TIF file, 1.6 MB. Copyright © 2021 Liang et al. 2021 Liang et al. https://creativecommons.org/licenses/by/4.0/ This content is distributed under the terms of the Creative Commons Attribution 4.0 International license.

**FIG 3:**
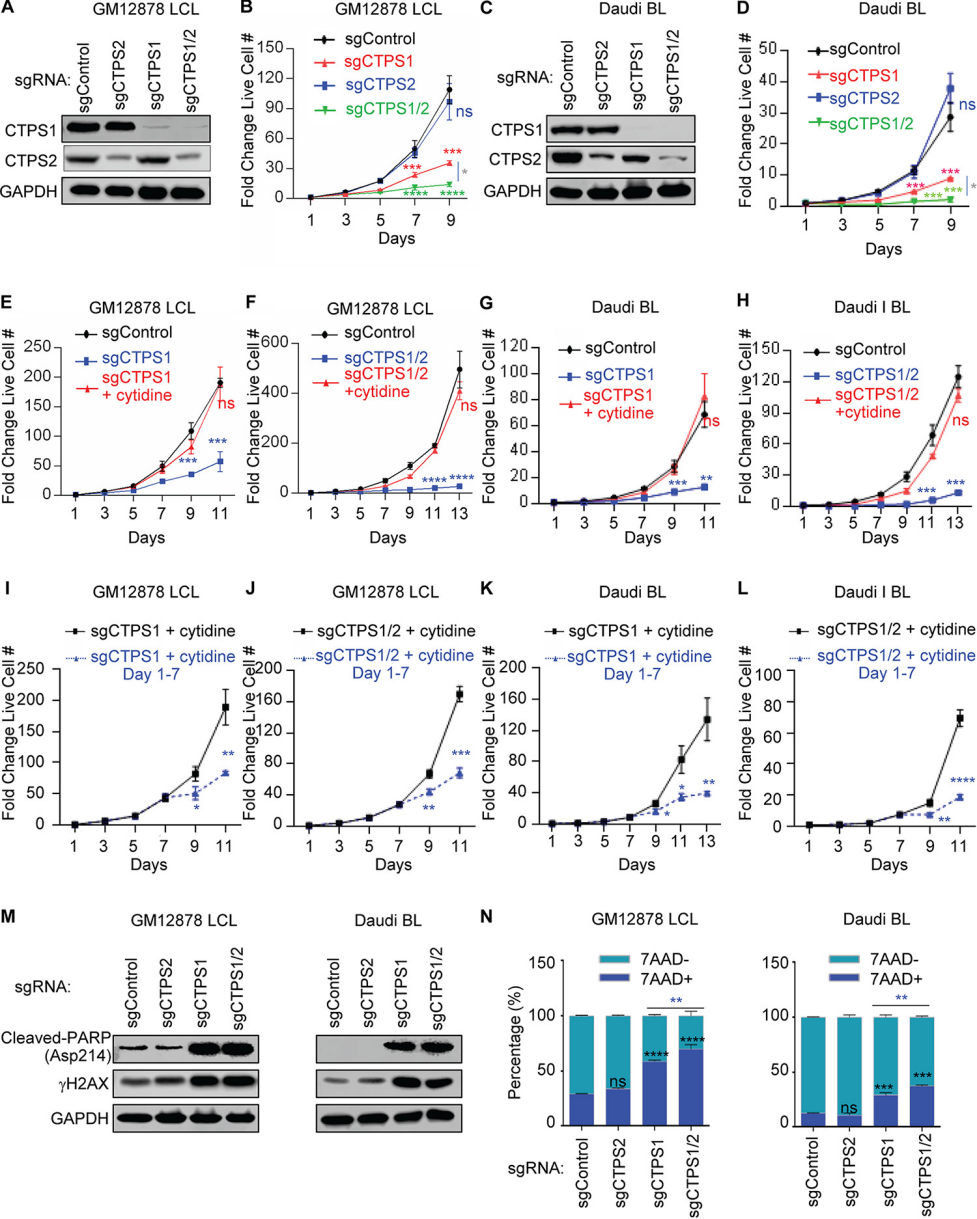
Partially redundant CTPS1/2 roles in EBV-transformed B cell growth and survival. (A) Immunoblot of WCL from GM12878 LCL on day 7 after expression of the indicated control, CTPS1, and/or CTPS2 sgRNAs. (B) Growth curve analysis of GM12878 LCL with control, CTPS1, and/or CTPS2 sgRNAs, as indicated. (C) Immunoblot of WCL from Daudi BL day 7 after expression of the indicated control, CTPS1, and/or CTPS2 sgRNAs. (D) Growth curve analysis of Daudi BL with control, CTPS1, and/or CTPS2 sgRNAs, as indicated. (E to H) Growth curve analysis of GM12878 LCL (E and F) or Daudi BL (G and H) that express control, CTPS1, or CTPS1 and CTPS2 sgRNAs as indicated, with 200 μM cytidine supplementation. (I to L) Growth curve analysis of GM12878 (I), GM11830 (J), Daudi (K), or Mutu I (L) with CTPS1 sgRNA expression, cultured in the presence of 200 μM cytidine from days 1 to 7 or throughout. (M) Immunoblot analysis of WCL from GM12878 (left) or Daudi (right) at day 7 after expression of the indicated sgRNAs. (N) Mean plus the SEM values of FACS 7-AAD values from GM12878 (left) or Daudi (right) expressing the indicated sgRNAs for 7 days from *n* = 3 replicates. Blots in panels A, C, and M are representative of *n* = 3 replicates. Growth curves were begun at 96 h after sgRNA expression and 48 h after puromycin selection. Growth curves show means plus the SEM fold change live cell numbers from *n* = 3 replicates. *, *P < *0.05; **, *P < *0.01; ***, *P < *0.001; ****, *P < *0.0001; ns, nonsignificant using one-way ANOVA with multiple comparisons.

To address this question, we next tested the effects of CTPS2 editing in LCLs and Burkitt cells, alone or in the context of CTPS1 depletion. In contrast to CTPS1 depletion, CTPS2 targeting did not significantly impair LCL or Burkitt B-cell growth or survival. However, concurrent CTPS1/2 targeting nearly completely abrogated LCL outgrowth, to a greater extent than depletion of CTPS1 alone (Fig. 3A to D; see also Fig. S3E and F). Cytidine supplementation rescued outgrowth of CTPS1- or CTPS1/2-deficient GM12878 LCLs or Daudi Burkitt cells (Fig. S3B-F), suggesting that CRISPR effects were on target and demonstrating that the cytidine salvage pathway was capable of restoring sufficient CTP pools. Interestingly, withdrawal of exogenous cytidine at day 7 of the proliferation assay rapidly blocked outgrowth of CTPS1- and CTPS1/2-deficient GM12878 and Daudi cells (Fig. 3I to L), further underscoring their acquired dependence on salvage CTP metabolism. CTPS1/2 depletion caused DNA damage, apoptosis induction and cell death in GM12878 and Daudi, as judged by immunoblotting for cleaved-PARP and γH2AX (Fig. 3M). 7-AAD uptake was higher following dual CTSP1/2 targeting (Fig. 3N).

### CTPS1 transcription upregulation in EBV-transformed B cells.

**FIG 4:**
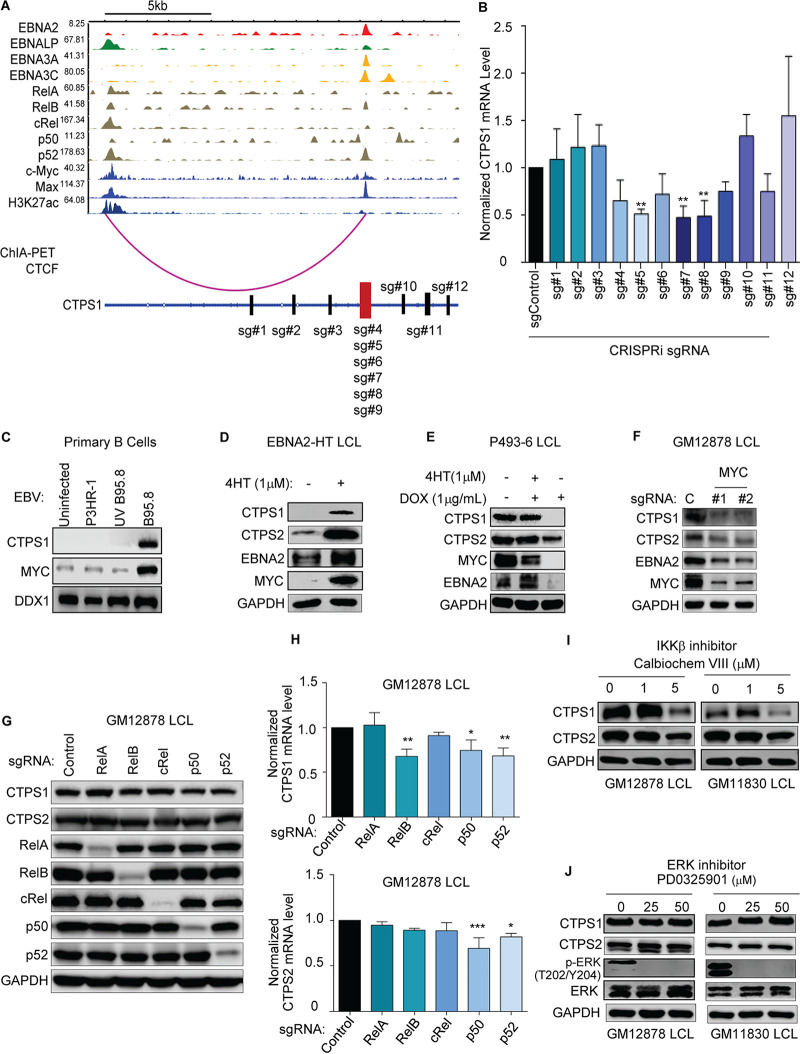
CTPS1 transcription regulation in EBV-infected B cells. (A) Chromatin immunoprecipitation-sequencing (ChIP-seq) tracks for the indicated transcription factors or histone 3 lysine 27 acetyl (H3K27Ac) at the LCL *CTPS1* locus. *y*-axis ranges are indicated for each track. Also shown below is LCL RNA Pol II ChIA-PET signal indicating a long-range DNA interaction between the *CTPS1* promoter region and an intronic site. A schematic diagram of the *CTPS1* locus shown below, with exons indicated by rectangles. CRISPR-i sgRNA targeting sites are shown. (B) Normalized CTPS1 mRNA levels in dCAS9-KRAB^+^ GM12878 expressing the indicated sgRNAs for CRISPR-i analysis for 5 days. CTPS1 mRNA level in cells with nontargeting control sgRNA was set to 1. (C) Immunoblot of WCL from primary human B cells that were either mock infected or infected with equal amounts of the nontransforming P3HR-1, UV-irradiated B95-8, or B95-8 EBV strain for 4 days. (D) Immunoblot analysis of samples prepared from 2-2-3 EBNA2-HT LCLs grown in the absence (EBNA2 nonpermissive) or presence (EBNA2 permissive) of 4HT (1 μM) for 48 h. (E) Immunoblot analysis of WCL from conditional P493-6 LCLs treated with 1 μg/ml doxycycline (DOX) to suppress exogenous *MYC* allele expression and/or with 1 μM 4HT to induce EBNA2 activity for 48 h, as indicated. (F) Immunoblot analysis of WCL from GM12878 LCL expressing control or independent *MYC*-targeting sgRNAs for 5 days, as indicated. (G) Immunoblot analysis of WCL from GM12878 LCL expressing the indicated sgRNA for 6 Days. (H) qPCR analysis of CTPS1 and CTPS2 mRNA levels in GM12878 LCL expressing the indicated sgRNAs for 7 days. Levels in cells with sgControl were set to 1. (I) Immunoblot of WCL from GM12878 or GM11830 LCLs treated with the indicated concentrations of the IKK antagonist Calbiochem VIII for 48 h. A concentration of 1 μM selectively blocks the canonical pathway IKKβ kinase. (J) Immunoblot of WCL from GM12878 or GM11830 LCLs treated with the ERK inhibitor PD0325901 at the indicated concentrations for 48 h. Blots in panels C, D, E, F, G, I, and J are representative of *n* = 3 experiments. Mean plus SEM values are shown in panels B and H from *n* = 3 replicates. *, *P < *0.05; **, *P < *0.01; ***, *P < *0.001 using one-way ANOVA with multiple comparisons.

The T-cell receptor and ERK pathways are important for rapid CTPS1 expression in activated T cells. Since pathways that control CTPS1 induction in EBV-transformed B cells remain unstudied, we investigated roles of EBV oncoproteins and key host targets. We initially used our and ENCODE published LCL ChIP-seq data sets to characterize occupancy of EBV nuclear antigens and EBV LMP1-activated NF-κB transcription factors (31–33). This analysis highlighted *CTPS1* promoter region occupancy by EBV transcription factor EBNA-LP, by multiple NF-κB transcription factor subunits, by c-MYC and MAX (Fig. 4A). Interestingly, EBNAs 2, LP, 3A, 3C, and NF-κB subunits RelB and p52 co-occupied an intergenic region ∼12 kb downstream from the *CTPS1* promoter. RNA pol II GM12878 Chromatin Interaction Analysis by Paired-End Tag Sequencing (ChIA-PET) analysis identifies a putative long-range interaction between Pol II bound to this *CTPS1* intragenic region and the *CTPS1* promoter (34).

To study the functional significance of the EBNA and NF-κB-bound *CTPS1* intragenic region in *CTPS1* transcription regulation, we used CRISPR-interference (CRISPR-i) (35). GM12878 LCLs with endonuclease dead Cas9 (dCas9) fused to a KRAB transcription repressor domain were transduced with lentiviruses that express control sgRNA versus one of the 12 sgRNAs indicated in Fig. 4A. In this manner, sgRNAs direct dCsa9-KRAB repressive activity to specific chromatin sites. Following puromycin selection, CRISPR-i effects on steady-state *CTPS1* mRNA abundance were quantified by real-time PCR. Four of the six sgRNAs targeting the putative enhancer significantly reduced CTPS1 mRNA levels. In contrast, none of the six sgRNAs targeting upstream or downstream control regions diminished CTPS1 mRNA (Fig. 4A and B). These results suggest that the EBV latency III growth program induces *CTPS1* induction through effects at the *CTPS1* promoter and at a downstream interacting region.

EBNA2 and EBNA-LP are the first EBV proteins expressed in newly infected cells. To gain insights into potential EBNA2 roles in EBV-driven *CTPS1* induction, we infected human peripheral blood CD19^+^ B cells, isolated by negative selection, with transforming B95.8 EBV, with UV-inactivated B95.8, or with nontransforming P3HR-1 EBV that lacks EBNA2 and part of the EBNA-LP open reading frames. Only infection by B95.8 induced MYC, a well characterized regulator of metabolism, as well as CTPS1 by 48 h of infection (Fig. 4C). These data suggest that EBNA2 and/or EBNA-LP, rather than an innate immune response to the incoming viral particle, are required for CTPS1 induction in newly infected B cells. We next used 2-2-3 LCLs with conditional EBNA2 expression, in which EBNA2 is fused to the ligand binding domain of a modified estrogen receptor that binds to 4-hydroxy-tamoxifen (4HT), to study EBNA2 roles in LCL CTPS1 expression. When grown under nonpermissive conditions in the absence of 4HT for 48 h, CTPS1 expression was rapidly lost, as was expression of EBNA2 target gene MYC (Fig. 4D). Thus, EBNA2, perhaps together with its host target genes, is important for EBV-driven CTPS1 induction.

To characterize putative CTPS1 induction roles of the key host EBNA2 target MYC, we used p493-6 LCLs, which carry conditional 4HT-controlled EBNA2 and tetracycline-repressed *MYC* alleles (36). EBNA2 inactivation by 4HT withdrawal again caused loss of CTPS1 and MYC expression in cells cultured with doxycycline to suppress exogenous *MYC* (Fig. 4E). Interestingly, induction of exogenous MYC expression by doxycycline withdrawal restored CTPS1 expression in the absence of EBNA2 activity (Fig. 4E). Similarly, MYC knockout in GM12878 blocked CTPS1 expression at a time point prior to induction of cell death (Fig. 4F), further supporting a key role for MYC in CTPS1 regulation and providing a potential route for CTPS1 induction in Burkitt cells. Interestingly, MYC KO reduced EBNA2 protein abundance (Fig. 4F).

The EBV-encoded CD40 mimic LMP1 activates the NF-κB canonical and noncanonical pathways to trigger nuclear translocation of the five NF-κB transcription factor subunits. Since GM12878 ChIP-seq identified *CTPS1* locus occupancy by all NF-κB subunits, we used CRISPR to test their individual roles in LCL CTPS1 expression (Fig. 4G). Depletion of RelB, p50 or p52 each reduced CTPS1 steady-state protein and mRNA levels (Fig. 4G and H). Since RelB forms heterodimers with either p50 or p52 in LCLs (31), this CRISPR analysis suggests the noncanonical NF-κB pathway supports CTPS1 expression in latency III B cells. In support, a IκB kinase (IKK)-β-selective antagonist did not significantly reduce CTPS1 protein levels at a dose where the canonical NF-κB pathway is selectively blocked. In contrast, the inhibitor impaired CTPS1 expression at a higher dose that also blocks the noncanonical NF-κB pathway (Fig. 4I). ERK inhibition by an antagonist that blunts T-cell CTPS1 expression (23) failed to reduce CTPS1 expression, in spite of LMP1’s ability to activate ERK (Fig. 4J). Thus, distinct mechanisms that control CTPS1 expression in EBV^+^ B cells versus T cells.

### EBV^+^ B cells use *de novo* and salvage UTP metabolism.

**FIG 5:**
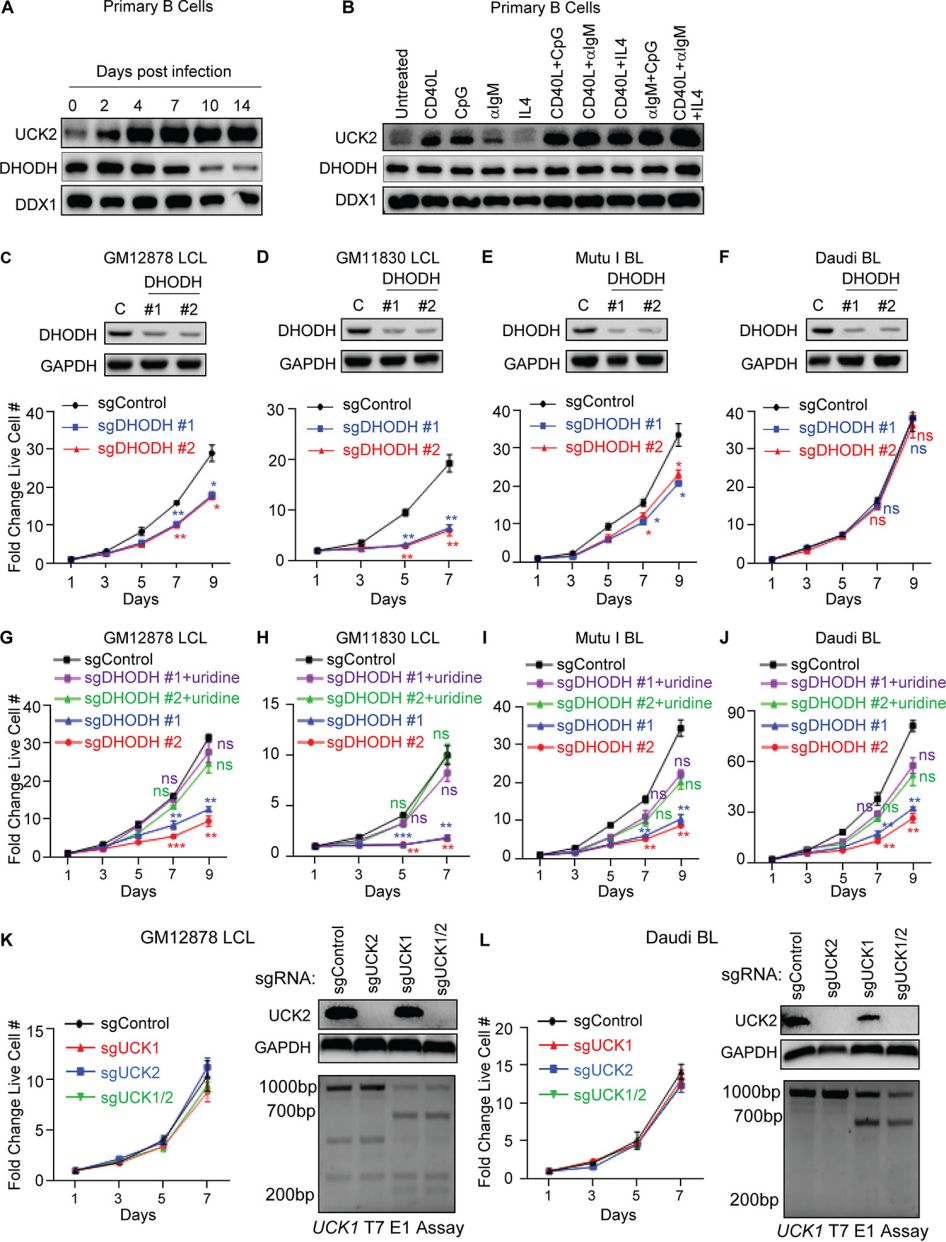
De novo pyrimidine synthesis enzyme DHODH and uridine salvage roles in EBV-transformed B-cell growth. (A) Immunoblot analysis of UCK2, DHODH and load-control DDX1 levels in whole-cell lysate (WCL) from human CD19^+^ peripheral blood B cells infected by the EBV B95.8 strain for the indicated days. (B) Immunoblot analysis of UCK1, DHODH, and DDX1 abundances in primary human CD19^+^ B cells stimulated for 24 h by CD40L (50 ng/ml), CpG (1 μM), μIgM (1 μg/ml), IL-4 (20 ng/ml), or combinations thereof, as indicated. (C to F) Immunoblot analysis (top) and growth curve analysis (bottom) of GM12878 (C), GM11830 (D), Mutu I (E), and Daudi (F) expressing nontargeting control or independent sgRNAs against *DHODH* in media containing 10% undialyzed FBS. WCLs were obtained at day 7 after sgRNA expression. (G to J) Growth curve analysis of GM12878 (G), GM11830 (H), Mutu I (I), and Daudi (J) expressing control or independent sgRNAs against DHODH. Cells were grown in media containing dialyzed FBS and supplemented with 200 μM uridine where indicated. (K) Growth curve analysis (left) and immunoblot of WCL or T7 endonuclease I (T7E1) assays of CRISPR editing of the *UCK1* locus (right) from GM12878 expressing the indicated sgRNAs. For this assay, UCK1 locus DNA flanking the Cas9 target site is PCR amplified from control and UCK1 edited cells, mixed, heated, and annealed. T7E1 cuts when annealed DNA contains a mismatch, resulting from Cas9 activity. (L) Growth curve analysis (left) and immunoblot of WCL or T7E1 assays of CRISPR editing of the *UCK1* locus (right) from Daudi cells expressing the indicated sgRNAs. Blots in panels A to F, K, and L are representative of *n* = 3 experiments. Mean plus SEM values are shown in panels C to L from *n* = 3 replicates. *, *P < *0.05; **, *P < *0.01; ***, *P < *0.001; ns, nonsignificant using one-way ANOVA with multiple comparisons.

Uracil monophosphate (UMP), a key precursor in *de novo* CTP synthesis, can be synthesized *de novo* from l-glutamine and l-aspartate by the sequential enzymatic activities of CAD, DHODH, and UMPS. UMP can also be produced by the salvage pathway, in which the kinases UCK1 or UCK2 phosphorylate imported uridine (Fig. 1A). Interestingly, UCK was originally identified as a B-cell factor that associates with EBNA3A, suggesting possible roles in EBV-driven B-cell proliferation (37). To gain insights into potential DHODH *de novo* versus UCK1/2 salvage roles in newly EBV-infected primary B cells, immunoblot analysis was performed on extracts from cells prior to and at five time points postinfection. At the protein level, UCK2 was robustly induced by day 4 postinfection, whereas DHODH expression was suppressed by day 10, despite each being induced on the mRNA level at this time point (Fig. 5A; see also Fig. S4A and B). By comparison, UCK1, UCK2, and DHODH mRNAs were upregulated by multiple primary B-cell stimuli, while again UCK2, but not DHODH, was upregulated on the protein level (Fig. 5B; see also Fig. S4C).

10.1128/mBio.01530-21.5 FIG S4 Profiling of EBV and immune receptor stimulation effects on UCK1, UCK2 and DHODH expression in primary B cells. (A) qPCR analysis of UCK1 and UCK2 mRNA levels in human peripheral blood CD19^+^ B cells at the indicated day post infection by B95.8 EBV at an MOI of 0.1. Day 0 values were set to 1. (B) UCK1, UCK2, and DHODH relative protein abundances from published analyses of tandem-mass-tag-based proteomic analysis at rest and at nine time points after EBV B95.8 infection of human peripheral blood CD19^+^ B cells at an MOI of 0.1 (28). (C) qPCR analysis UCK1, UCK2 or DHODH mRNA levels in human peripheral blood CD19^+^ B cells stimulated for 24 h by Mega-CD40L (50 ng/ml), CpG (1μM), αIgM crosslinking (1 μg/ml), IL-4 (20 ng/ml), or combinations thereof, as indicated. Mean plus SEM values are shown in panels A to C from *n* = 3 replicates. *, *P < *0.05; **, *P < *0.01; ***, *P < *0.001; ****, *P < *0.0001; ns, nonsignificant using one-way ANOVA with multiple comparisons. Download FIG S4, TIF file, 1.8 MB. Copyright © 2021 Liang et al. 2021 Liang et al. https://creativecommons.org/licenses/by/4.0/ This content is distributed under the terms of the Creative Commons Attribution 4.0 International license.

DHODH inhibition prevents EBV lymphomas in a humanized mouse model (24). We next used CRISPR to test the effects of DHODH depletion on LCL or Burkitt growth and survival. In media containing undialyzed fetal bovine serum (FBS), which contains uridine (Fig. 1A), independent DHODH sgRNAs impaired proliferation of both LCLs and of Mutu I, but not Daudi Burkitt cells (Fig. 5C to F). To more closely examine the ability of EBV-transformed B cells to use uridine as a substrate for *de novo* CTP synthesis, control and DHODH edited LCL and Burkitt lymphoma (BL) cells were grown with dialyzed FBS lacking uridine and thereby blunting salvage metabolism. In this context, DHODH depletion more strongly inhibited proliferation, which was rescued by uridine add-back (Fig. 5G to J; see also Fig. S5A to D). Thus, LCLs can use amino acid *de novo* or uridine salvage for CTP synthesis (Fig. 1A).

10.1128/mBio.01530-21.6 FIG S5 Analysis of DHODH, UCK1/2 and uridine salvage metabolism roles in EBV-transformed B-cell growth. (A and B) Growth curve analysis of GM11830 (A) or Mutu I (B) expressing control or DHODH sgRNAs and puromycin selected for 48 h and then grown in media containing uridine-depleted 10% dialyzed FBS, in the absence or presence of 200 μM uridine supplementation. Immunoblot analysis of WCL from cells with the indicated sgRNAs are shown to the right. (C) FACS analysis of 7-AAD abundances in GM12878, GM11830, Mutu I, or Daudi expressing the indicated sgRNAs for 7 days and grown in media containing uridine-depleted, dialyzed FBS in the absence or presence of 200 μM uridine supplementation. (D) Mean plus SEM values of FACS analysis of *n* = 3 replicates, as in panel C. (E and F) Growth curve analysis (left) and immunoblot of WCL for UCK2 and GAPDH load control or *UCK1* locus T7E1 assay results (right) of GM11830 (E) or Mutu cells (F) expressing the indicated sgRNAs. Blots in panels A, B, E, and F are representative of *n* = 3 experiments. Mean plus SEM values are shown in panels A, B, D, E, and F from *n* = 3 replicates. *, *P < *0.05; **, *P < *0.01; ***, *P < *0.001; ns, nonsignificant using one-way ANOVA with multiple comparisons. Download FIG S5, TIF file, 1.7 MB. Copyright © 2021 Liang et al. 2021 Liang et al. https://creativecommons.org/licenses/by/4.0/ This content is distributed under the terms of the Creative Commons Attribution 4.0 International license.

We next used CRISPR to test the effects of UCK1 and/or UCK2 depletion on EBV-transformed B-cell proliferation. Successful CRISPR editing of UCK2 was confirmed by immunoblotting, whereas UCK1 editing was confirmed by T7 E1 assay, since we were unable to find a suitable immunoblot antibody. Unexpectedly, depletion of UCK1, UCK2, or UCK1/2 did not significantly impair GM12878 LCL or Burkitt cell proliferation (Fig. 5K and L; see also Fig. S5E). These results suggest that LCLs and Burkitt cells can synthesize sufficient CTP *de novo* when glutamine and aspartate are available but that they are nonetheless able to switch to uridine salvage when necessary.

### CTPS1 roles in EBV lytic DNA replication.

**FIG 6:**
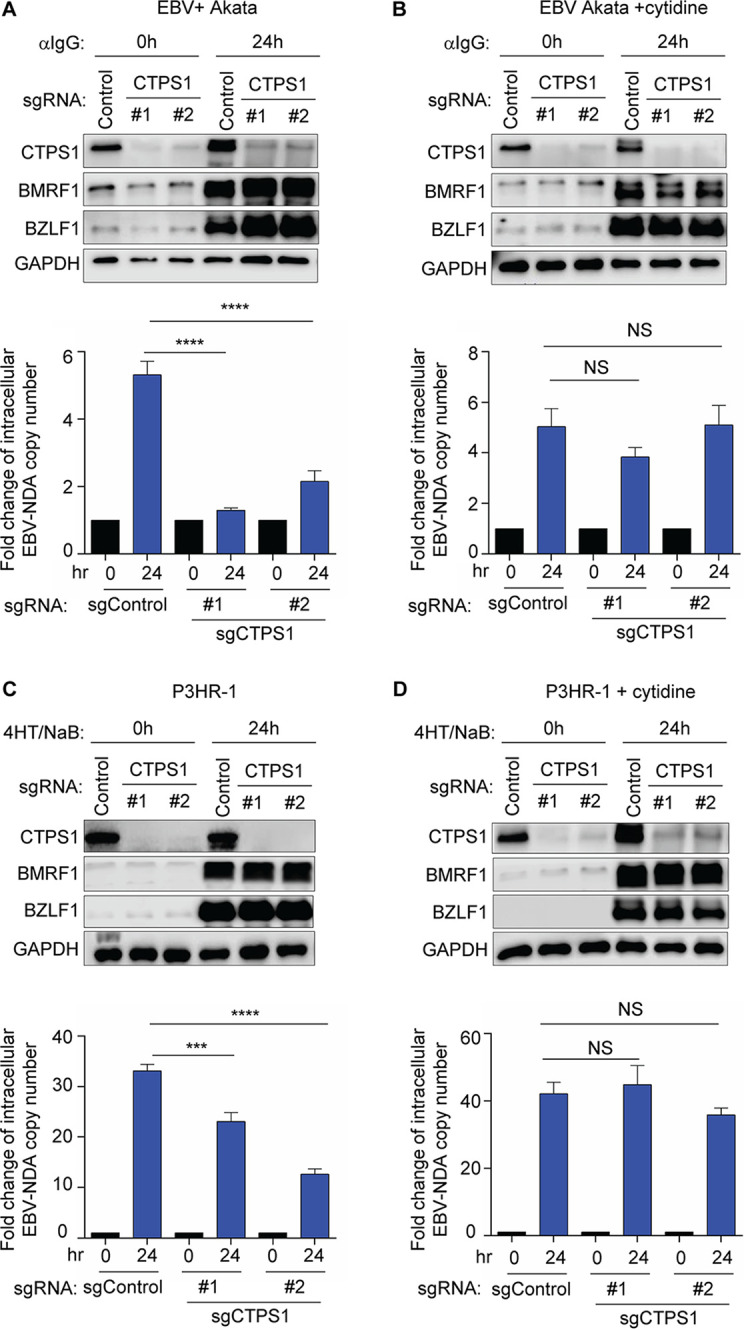
Key CTPS1 role in EBV lytic DNA replication. (A) Immunoblot analysis of WCE from EBV^+^ Akata BL cells expressing the indicated sgRNAs for 96 h and mock induced or induced for lytic replication by αIgG (10 μg/ml) cross-linking for 24 h. Blots for immediate early BZLF1 and early BMRF1 lytic antigens, CTPS1, and GADPH are shown. Shown below are EBV genome copy numbers determined by qPCR analysis. Values for mock-induced cells with sgControl were set to 1. Relative intracellular EBV DNA copy numbers were determined by qPCR using primers to the *BALF5* gene and were normalized for input cell number using primers to *GAPDH*. (B) Immunoblots and qPCR analysis of EBV genome copy number as in panel A, from cells grown in the presence of 200 μM cytidine rescue. (C) Immunoblot analysis of WCE from Cas9^+^ P3HR-1 Burkitt cells expressing a conditional 4-HT regulated BZLF1 allele and the indicated sgRNAs, mock induced or induced for EBV lytic reactivation by addition of 400 nM 4HT and 500 μM sodium butyrate for 24 h. sgRNAs were expressed for 6 days. Blots for EBV-encoded wild-type BZLF1 and BMRF1, as well as for CTPS1 and GADPH, are shown. Shown below are qPCR analysis of EBV genome copy number, as in panel A. (D) Immunoblots and qPCR analysis of EBV genome copy number as in panel C from cells grown in the presence of 200 μM cytidine rescue. Blots in panels A to D are representative of *n* = 3 replicates. Mean plus SEM values from *n* = 3 of biologically independent replicates are shown in panels A to D. ****, *P < *0.0001; ***, *P < *0.001; NS, nonsignificant using one-way ANOVA with multiple comparisons.

Knowledge remains incomplete of how EBV remodels CTP synthesis pathways in support of the B-cell lytic cycle, where the viral immediate early transcription factors BZLF1 and BRLF1 induce early genes responsible for viral DNA replication and subsequently expression of viral late genes. Lytic replication results in synthesis of hundreds to thousands of EBV ∼170-kb genomes by the viral DNA polymerase (38). To test *de novo* cytidine roles in EBV lytic replication, control or *CTPS1* targeting sgRNAs were expressed in EBV^+^ Akata Burkitt cells, a model for EBV lytic replication. After CRISPR editing, lytic replication was induced by B-cell receptor cross-linking for 24 h. Interestingly, while CTPS1 depletion did not appreciably affect expression of the immediate early gene BZLF1 or early gene BMRF1, production of lytic EBV genomes was significantly impaired by independent CTPS1 sgRNAs (Fig. 6A). Lytic EBV genome replication was rescued by cytidine add-back, suggesting that either the *de novo* or salvage CTP pathways can support the viral lytic cycle (Fig. 6B). Similar results were obtained in P3HR-1 Burkitt cells, in which lytic replication was induced by a conditional *BZLF1* allele together with sodium butyrate (Fig. 6C and D). In agreement with EBV genome copy number assays, CTPS1 KO significantly reduced the amount of infectious EBV produced upon Ig cross-linking, as judged by the green Raji unit assay (see Fig. S6). Importantly, cytidine supplementation rescued green Raji units in cells expressing either of two independent CTPS1 sgRNAs, indicative of on-target effects.

10.1128/mBio.01530-21.7 FIG S6 Green Raji unit analysis of EBV infectious units from wild-type versus CTPS1 KO cells, in the absence or presence of cytidine supplementation. Lytic replication was triggered by Ig cross-linking (10 mg/ml) for 24 h in Cas9^+^ Akata Burkitt cells expressing control or CTPS1 sgRNAs, in the absence or presence of 200 μM cytidine supplementation, as indicated. Akata cells contained EBV BAC genomes with a GFP marker to enable tracking of target cell infection. Filtered supernatants were then added onto Raji target B cells at the indicated serial dilution. The percentages of GFP^+^ Raji cells, indicative of infectious EBV units, were measured by FACS 72 h later. Mean plus SEM values are shown in panels A, B, D, E, and F from *n* = 3 replicates. *, *P < *0.05; **, *P < *0.01; ***, *P < *0.001; ns, nonsignificant using one-way ANOVA with multiple comparisons. Download FIG S6, TIF file, 0.8 MB. Copyright © 2021 Liang et al. 2021 Liang et al. https://creativecommons.org/licenses/by/4.0/ This content is distributed under the terms of the Creative Commons Attribution 4.0 International license.

## DISCUSSION

The ability of B and T lymphocytes to rapidly switch from a quiescent state into rapidly growing blasts upon antigen receptor and coreceptor stimulation underlies adaptive immunity. CTPS1 is highly induced by T-cell stimulation through the ERK pathway and is necessary for rapid T-cell proliferation and the control of acute EBV infection (23). However, with hypomorphic CTPS1 activity, patients often exhibit severe infectious mononucleosis, chronic active EBV and EBV^+^ CNS B-cell lymphomas. Since EBV relies on host metabolism pathways to convert newly infected cells into hyperproliferating blasts and to support lytic replication, these clinical observations raise the question of how individuals with CTPS1 deficiency exhibit elevated EBV viral loads and frequent B-cell lymphomas, despite hypoactivity of a key CTP generation enzyme, yet high CTP demands of B-cell growth (16, 23). Further adding to this question, classic studies indicate that CTP synthase activity is frequently upregulated in cancer (39).

We therefore tested the effects of CTPS1, CTPS2, and CTP salvage pathways to support EBV-infected B-cell growth, survival, and EBV lytic replication. EBV-mediated B-cell growth transformation proceeds through a genetically encoded process that proceeds through Burkitt-like hyperproliferation and lymphoblastoid phases, whose physiology resembles the Burkitt and LCL cell lines used in this study (28, 39). LCLs are an established tissue culture model for immunoblastic lymphomas of immunosuppressed hosts, including CNS lymphoma. The results presented here suggest that while CTPS1 activity is important for EBV-transformed B-cell proliferation, EBV-infected B cells can also utilize CTPS2 activity and cytidine salvage metabolism to support CTP synthesis for DNA, RNA, phosphatidylcholine, and phosphatidylserine needs. While knowledge of cytidine concentrations in tonsil and lymphoid tissues remains limited, robust deoxycytidine kinase activity is evident in human tonsil tissue (40).

We speculate that in patients with CTPS1 deficiency, CTPS2 compensation is insufficient for cell-mediated control of EBV infection, but is nonetheless able to support EBV^+^ B-cell growth, together with hypomorphic CTPS1. Given our findings that CTPS2 supports proliferation of EBV-transformed CTPS1-deficient B cells, we speculate that CTPS2 plays important roles in the CNS lymphomas observed clinically. Consequently, CTPS1/2 may serve as a therapeutic synthetic lethal target in this setting, for instance if cytidine replacement fails to restore antitumor immunity. While our quantitative proteomic analyses identified that CTPS1 is approximately four times more abundant than CTPS2 in primary human B cells undergoing EBV-mediated growth transformation (28), EBV-infected lymphomas may compensate for CTPS1 deficiency by increasing CTPS2 levels and/or activity *in vivo*. It would be of interest to test this hypothesis through RNA-seq and enzymatic profiling of CNS lymphoma samples.

CTPS1 and CTPS2 are each highly regulated at the posttranslational level, including by assembly into filaments called cytoophidium that regulate catalytic activity (41–43). Whether cytoophidium form in EBV-infected B cells generally or with CTPS1 deficiency is unknown. Furthermore, cryoelectron microscopy studies recently identified a novel filament-based mechanism of CTPS2 regulation that stabilizes nucleotide levels (44). Filaments containing CTPS1/2 and IMPDH, the rate-limiting enzyme in *de novo* GTP synthesis, have also been described (45) and may play important roles in EBV-infected B-cell metabolism.

CTP salvage pathways may also play important roles in support of pathogenic B-cell roles in CTPS1-deficient patients. Cytidine is imported into the CNS by nucleoside transporters located at the blood-brain barrier (46). That CTPS1/2-deficient LCL growth could be restored by cytidine supplementation highlights the potentially important role of the CTP salvage pathway in B cells with the viral latency III program observed in CNS lymphoma. Interestingly, since oral intake may alter CNS pyrimidine levels (46), dietary cytidine and uridine restriction could potentially have antineoplastic effects in CTPS1-deficient patients with CNS lymphoma.

Patients with CTPS1 deficiency often have elevated EBV viral loads. In principle, this result may result from latent or lytic states with impaired T-cell surveillance (47). However, we note that most EBV is cell associated and that whole blood viral load assays frequently used in the clinic likely detect latent EBV genomes resident in B cells or released from dying cells. Nonetheless, viral lytic gene expression has been associated with EBV lymphomagenesis in animal models (48–50). Interestingly, the DHODH chemical inhibitor teriflunomide blocks EBV lytic gene expression and DNA replication (24). However, we found that CTPS1 knockout instead reduces EBV lytic DNA replication, without impairing immediate early and early gene expression. Lytic genome replication is important for EBV late gene expression but not for that of immediate early or early genes. Also consistent with a potential role in supplying CTP for lytic genome synthesis, cytidine supplementation rescued EBV lytic DNA replication, newly identifying their ability to utilize cytidine salvage metabolism. Collectively, our data support a model in which EBV genome copy numbers are elevated in CTPS1-deficient patients as a result of an expanded pool of latently EBV-infected cells, whose EBV-driven oncogenic growth is not properly kept in check by T-cell surveillance.

**FIG 7:**
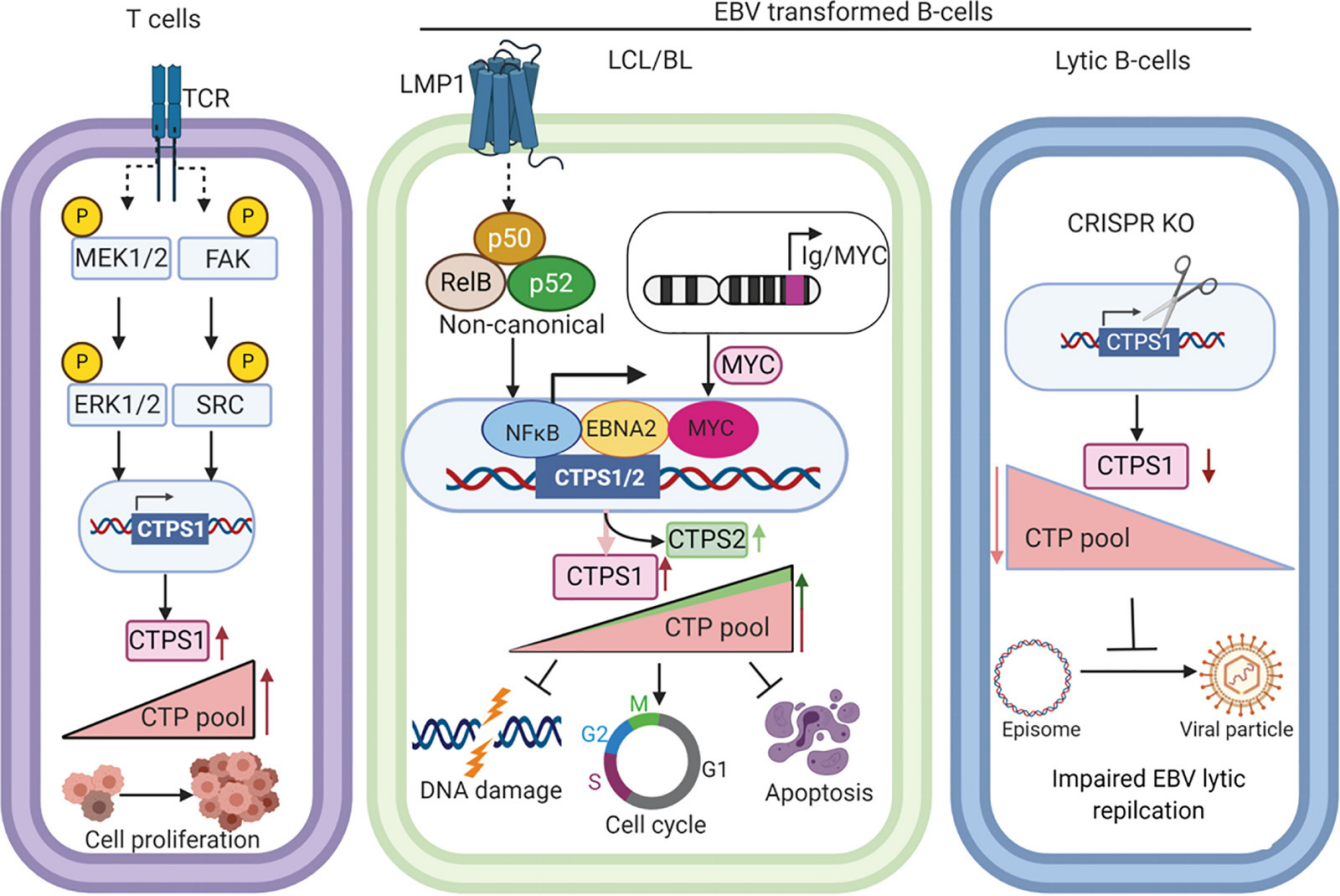
Schematic model of CTPS1 roles in T versus EBV-infected B cells. T-cell receptor stimulation induces CTPS1 expression, at least in part through the ERK and SRC pathways. In contrast, EBV induces CTPS1 in LCLs through EBNA2-, MYC-, and LMP1-activated noncanonical NF-κB pathway, whereas hyperexpressed MYC has key roles in Burkitt CTPS1 induction. CTPS1 and CTPS2 activity together supply CTP pools for EBV-transformed B cells to prevent nucleotide imbalance, DNA damage, cell cycle arrest, and apoptosis. CTPS1 depletion also impairs EBV lytic DNA replication.

CTP levels increase in EBV-transforming B cells, though to lower magnitude than other deoxynucleotide triphosphates (51). Feedback and allosteric regulation renders CTPS1 activity sensitive to the levels of all four nucleotides (22, 52). EBNA2 and MYC are important regulators of EBV-driven one-carbon metabolism and purine nucleotide synthesis in newly infected cells (28). Thus, their roles in CTPS1 induction likely coordinates pools of all four deoxynucleotides to avoid imbalances and DNA damage. Indeed, CRISPR knockout of CTPS1 resulted in H2AX phosphorylation. While NF-κB is not required for initial EBV-driven B-cell growth (53), it is essential for LCL growth and survival, where we found a noncanonical NF-κB role in CTPS1 expression. Our findings also suggest that hyper-active MYC activity is also sufficient to drive CTPS1 expression and likely supports CTPS1 expression in Burkitt cells. In contrast, ERK and SRC have obligatory roles in robust CTPS1 upregulation upon T-cell activation that are critical for T-cell expansion (Fig. 7). Notably, MYC has key roles in *de novo* pyrimidine nucleotide synthesis (54).

In summary, these studies highlight that multiple CTP synthesis pathways are operative in EBV-infected B cells and that each can play important roles in EBV transformed B-cell growth and lytic DNA replication. Collectively, these results highlight CTP synthesis pathways as important therapeutic targets in EBV-driven lymphoproliferative disorders.

## MATERIALS AND METHODS

### Reagents.

The following reagents or chemicals were used in this study: MEGA-CD40L (Enzo Life Sciences catalog no. ALX-522-110-C010, 50 ng/ml), CpG (IDT, TCGTCGTTTTGTCGTTTTGTCGTT, 1 μM), αIgM (Sigma, catalog no. 10759, 1 mg/ml), IL-4 (R&D Systems, 204-IL-050, 20 ng/ml), αIgG (Agilent, A042402-2), cytidine (Sigma, C122106, 200 mM), uridine (Sigma, U3003, 200 mM), 4-hydroxy tamoxifen (4HT; Sigma, H7904-25MG), doxycycline (Sigma, D9891-1G), sodium butyrate (ALFA AESAR AAA1107922), IKK inhibitor VIII (Calbiochem, catalog no. 401487), and MAPK/ERK inhibitor PD0325901 (Sigma, PD0325901). For rescue experiments, cytidine (200 μM) and uridine (200 μM) were added 2 days after puromycin selection for the indicated periods.

### Cell lines, culture, and growth curve analysis.

293T were purchased from ATCC and cultured in Dulbecco modified Eagle medium (Gibco) with 10% FBS (Thermo Fisher). All B cells were cultured in RPMI 1640 (Invitrogen) supplemented with 10% standard FBS and penicillin-streptomycin in a humidified incubator at 37°C and at 5% CO_2_. For assays with dialyzed serum, 10% dialyzed FBS (Thermo Fisher, 26400044) was used.

P3HR-1, Daudi, EBV^+^ Akata, and EBV^–^ Akata cells were previously obtained from Elliott Kieff. GM12878 and GM11830 were purchased from Coriell. Mutu I was obtained from Jeff Sample. 2-2-3 EBNA2HT LCLs with a conditional EBNA2 allele were obtained from Bo Zhao and Elliott Kieff. 2-2-3 LCLs express EBNA2 fused to a modified estrogen receptor 4HT-binding domain. In the presence of 4HT, the EBNA2HT allele localizes to the nucleus and is active, but upon 4HT withdrawal it is redistributed to the cytosol, where it is destabilized. 2-2-3 LCLs were maintained in the presence of 1 μM 4HT. To remove 4HT, cells were washed five times with 4HT-free media, including two incubations for 30 min, and then reseeded at 100,000 cells per ml in media with or without 4HT, as indicated. Cells were then grown for 48 h and harvested for RNA extraction and cell lysate preparation. The conditional P493-6 LCLs were a gift from Micah Luftig (Duke University). P493-6 cells have a conditional EBNA2HT allele similar to 2-2-3 LCLs and also have a TET-Off exogenous MYC allele. In the absence of tetracyclines, high-level exogenous MYC is induced. P493-6 cells were maintained continuously in the absence of 4HT or doxycycline to induce a BL-like state of high MYC expression. To grow in the lymphoblastoid cell state (intermediate MYC), P493-6 cells were grown in the presence of both 1 μM 4HT and doxycycline. To induce a low MYC state and consequently G_1_ growth arrest, cells were grown in the absence of 4HT and presence of 1 μM doxycycline. After 48 h of growth in any of these conditions, cells were collected for lysate preparation. All cell lines used for CRISPR editing in this study stable express Streptococcus pyogenes Cas9 gene. All cells were routinely confirmed to be mycoplasma negative by a Lonza MycoAlert assay (Lonza).

### Primary human B-cell isolation and culture.

Platelet-depleted venous blood obtained from the Brigham and Women’s Hospital bank were used for primary human B cell isolation, following our institutional guidelines for discarded and deidentified samples. RosetteSep (Stem Cell Technologies, catalog no. 15064) and EasySep negative isolation kits (Stem Cell Technologies, catalog no. 19054) were used sequentially to isolate CD19^+^ B cells with the following modifications made to the manufacturer’s protocols. For RosetteSep, 40 μl of antibody cocktail was added per ml of blood and then layered onto Lymphoprep density medium for centrifugation. For EasySep, 10 μl of antibody cocktail was added per ml of B cells, followed by 15 μl of magnetic bead suspension per ml of B cells. After negative selection, plasma membrane CD19 was used to confirm B cell purity. For most experiments, cells were cultured in RPMI 1640 (Invitrogen) supplemented with 10% standard FBS and penicillin-streptomycin. Cells were cultured in a humidified incubator at 37°C and at 5% CO_2_.

### CRISPR/Cas9 gene editing.

CRISPR/Cas9 knockout was performed as previously described (55). Briefly, Broad Institute pXPR-011 GFP-targeting or pXPR-515 control sgRNA, Avana or Brunello library sgRNAs, as listed in Table S1, were cloned into lentiGuide-Puro (Addgene, catalog no.52963) or pLenti SpBsmBI sgRNA Hygro (Addgene, catalog no. 62205). sgRNA-expressing lentiviruses were produced by 293T transfection. Media was exchanged to RPMI–10% FBS at 24 h posttransfection. Lentiviruses were harvested at 48 and 72 h posttransfection. Virus supernatant was filtered through a 0.45-μm syringe filter. B cells with stable Cas9 expression were transduced and selected with puromycin 3 μg/ml for 48 h or with hygromycin at 200 μg/ml for the initial 4 days and then at 100 μg/ml thereafter. CRISPR editing was confirmed by immunoblot or T7 endonuclease 1 (T7E1) mismatch detection assay, as indicated. Where an appropriate antibody for the target gene product could not be obtained, the T7 endonuclease I assay was instead used to confirm CRISPR editing using the primers listed in Table S2 in the supplemental material. Briefly, genomic DNA was collected from control and target gene knockout cells using a DNeasy blood and tissue kit from Qiagen according to the recommended protocol. T7E1 assays were conducted following the NEB EnGen Mutation Detection kit protocol (E3321; New England Biolabs). Briefly, Cas9-targeted regions were PCR amplified from genomic DNA harvested from cells expressing sgControl or specific sgRNAs. PCR products were purified by 2% agarose gel electrophoresis and then by purified by using a QIAquick gel extraction kit (Qiagen). A mixture containing PCR products amplified from control cells (500 ng) or from control cells and EBV genome sgRNA targeted cells (250 ng) were supplemented with 1 ml of NEB2 buffer, heated to 95 C for 10 min, and then cooled to 4°C at a cooling rate of 0.1°C/s, using a thermocycler. Subsequently, 0.5 ml of the T7E1 enzyme (250 U/ml of final concentration; New England Biolabs) was added. Restriction enzyme digests were incubated at 37°C for 30 min and subsequently separated on a 2% agarose gel.

10.1128/mBio.01530-21.8 TABLE S1 Sequences of sgRNAs used for CRISPR editing and CRISPR-i. Download Table S1, DOCX file, 0.01 MB. Copyright © 2021 Liang et al. 2021 Liang et al. https://creativecommons.org/licenses/by/4.0/ This content is distributed under the terms of the Creative Commons Attribution 4.0 International license.

10.1128/mBio.01530-21.9 TABLE S2 T7EI assay and RT-qPCR primers used in this study. Download Table S2, DOCX file, 0.01 MB. Copyright © 2021 Liang et al. 2021 Liang et al. https://creativecommons.org/licenses/by/4.0/ This content is distributed under the terms of the Creative Commons Attribution 4.0 International license.

### (i) CRISPR-i.

CRISPR-i sgRNAs were designed with online tools from Benchling (San Francisco, CA). sgRNAs for CRISPR-i were cloned into lentiGuide-Puro (catalog no. 52963; Addgene). Lentiviruses were prepared by transfecting HEK293T cells and used to transduce LCLs stably expressing a dCas9-KRAB fusion protein for CRISPR-i (35).

### (ii) Growth curve analysis.

Cells were normalized to the same starting concentration. Cell numbers were then quantified using the CellTiter-Glo assays at the indicated times.

### (iii) Statistical analysis.

The data are presented as means ± the standard errors of the means (SEM). Data were analyzed using analysis of variance (ANOVA) with Sidak’s multiple-comparison test or two-tailed paired Student *t* test with Prism7 software. For all statistical tests, a *P* cutoff value of <0.05 was used to indicate significance.

### RT-PCR analysis.

Total RNA was harvested from cells using RNeasy minikit (Qiagen, catalog no. 27106). Genomic DNA was removed by using the RNase-Free DNase set (Qiagen, catalog no. 79254). RNA was reversed transcribed by iScript reverse transcription supermix (Bio-Rad, catalog no. 1708841). qRT-PCR was performed using Power SYBR green PCR mix (Applied Biosystems, catalog no. 4367659) on aCFX96 Touch real-time PCR detection system (Bio-Rad), and data were normalized to internal control 18S. The relative expression was calculated using the 2^–ΔΔ^*^CT^* method. All samples were run in technical triplicates, and at least three independent experiments were performed. The primer sequences used are listed in Table S2.

### Quantification of EBV genome copy number.

To measure intracellular viral DNA, total genomic DNA was extracted from 2 × 10^6^ BL cells using the Blood & Cell culture DNA Max kit (Qiagen, catalog no. 13362). Extracted DNA was further diluted to 10 ng/μl and subjected to qPCR targeting the BALF5 gene. Standard curves were made by serial dilution of a pHAGE-BALF5 miniprep DNA at 25 ng/μl. Viral DNA copy number was calculated by inputting sample *C_q_* values into the regression equation dictated by the standard curve and normalized by GAPDH.

### Quantitation of EBV infectious virion levels by green Raji unit assay.

To quantitate levels of infectious EBV produced by wild-type versus CTPS1 KO Akata Burkitt cells, EBV lytic replication was induced by αIgG (10 μg/ml). At 3 days posttransfection, supernatants were collected and filtered through 0.8-μm-pore-size filters. Raji cells (1 × 10^5^) were infected with serial dilutions of virus stocks, incubated at 37°C in 24-well plates, and cultured for 3 days to allow for expression of green fluorescent protein (GFP), driven by the Akata EBV BAC GFP marker. At 24 h after Raji cell infection, the medium was exchanged to fresh RPMI–10% FCS. At 48 h postinfection, tetradecanoyl phorbol acetate and sodium butyrate were added to the cells at final concentrations of 20 ng/ml and 3 mM, respectively. At 72 h postinfection, the percentages of GFP^+^ cells were determined by fluorescence-activated cell sorting (FACS).

10.1128/mBio.01530-21.1 TEXT S1 Supplemental methods. Download Text S1, PDF file, 0.1 MB. Copyright © 2021 Liang et al. 2021 Liang et al. https://creativecommons.org/licenses/by/4.0/ This content is distributed under the terms of the Creative Commons Attribution 4.0 International license.
