# Supplementary figures and images for: Epstein-Barr Virus Induced Cytidine Metabolism Roles in Transformed B-Cell Growth and Survival
Source: mBio. 2021 Jul 20;12(4):e01530-21. doi: 10.1128/mBio.01530-21 (PMC8406234; doi:10.1128/mBio.01530-21)

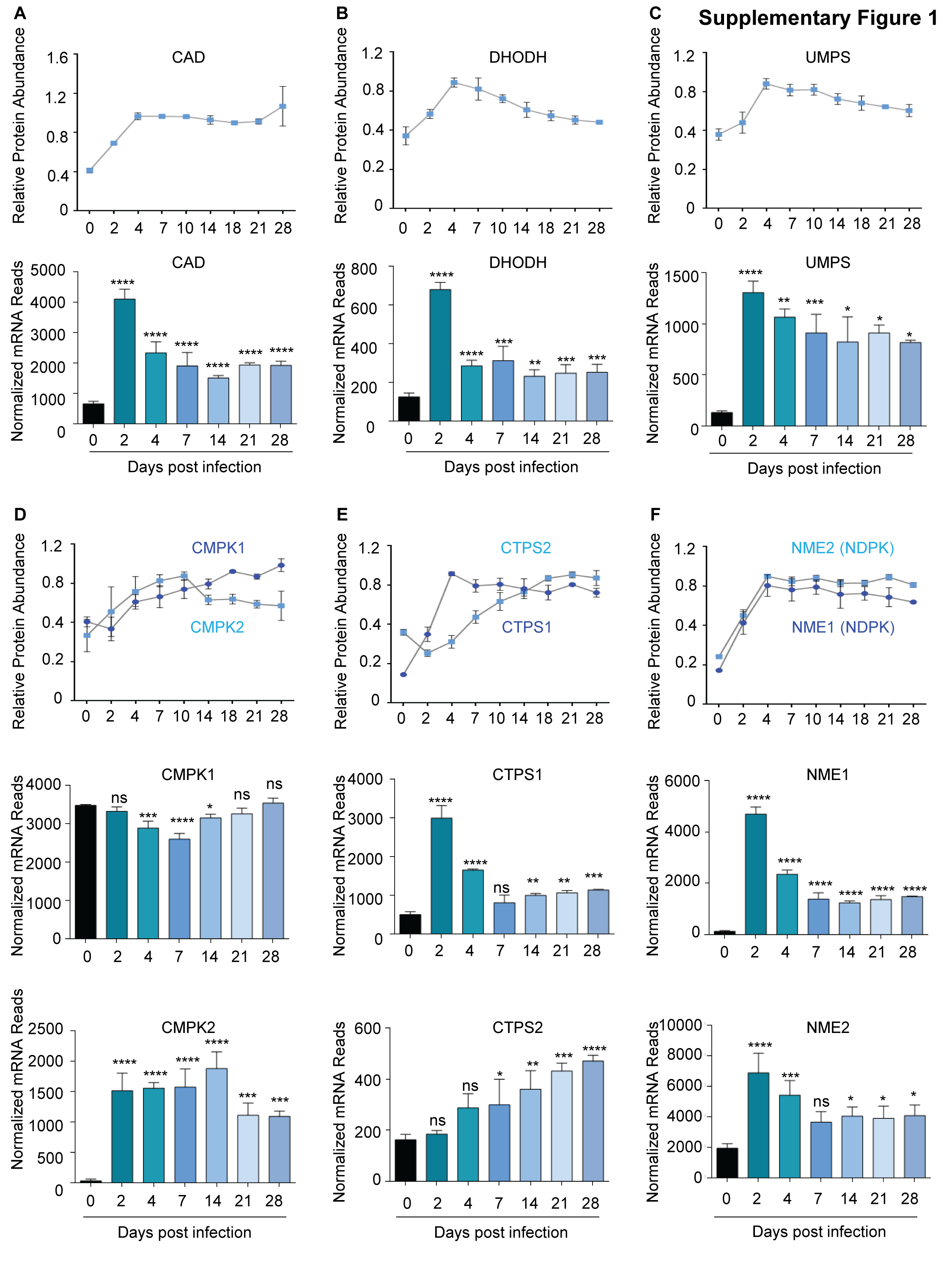

Supplement: FIG S1 [file mbio.01530-21-sf001.tif]

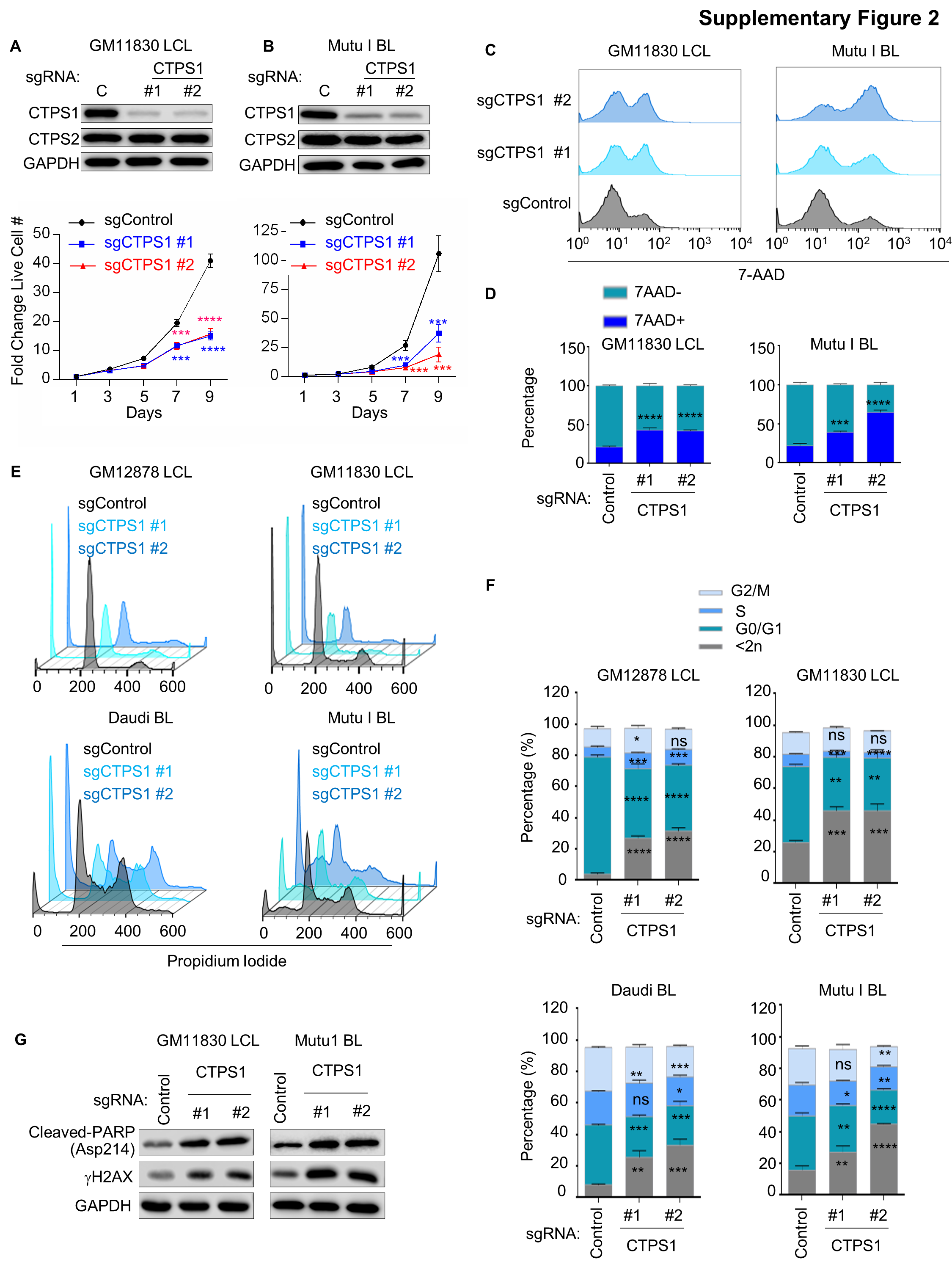

Supplement: FIG S2 [file mbio.01530-21-sf002.tif]

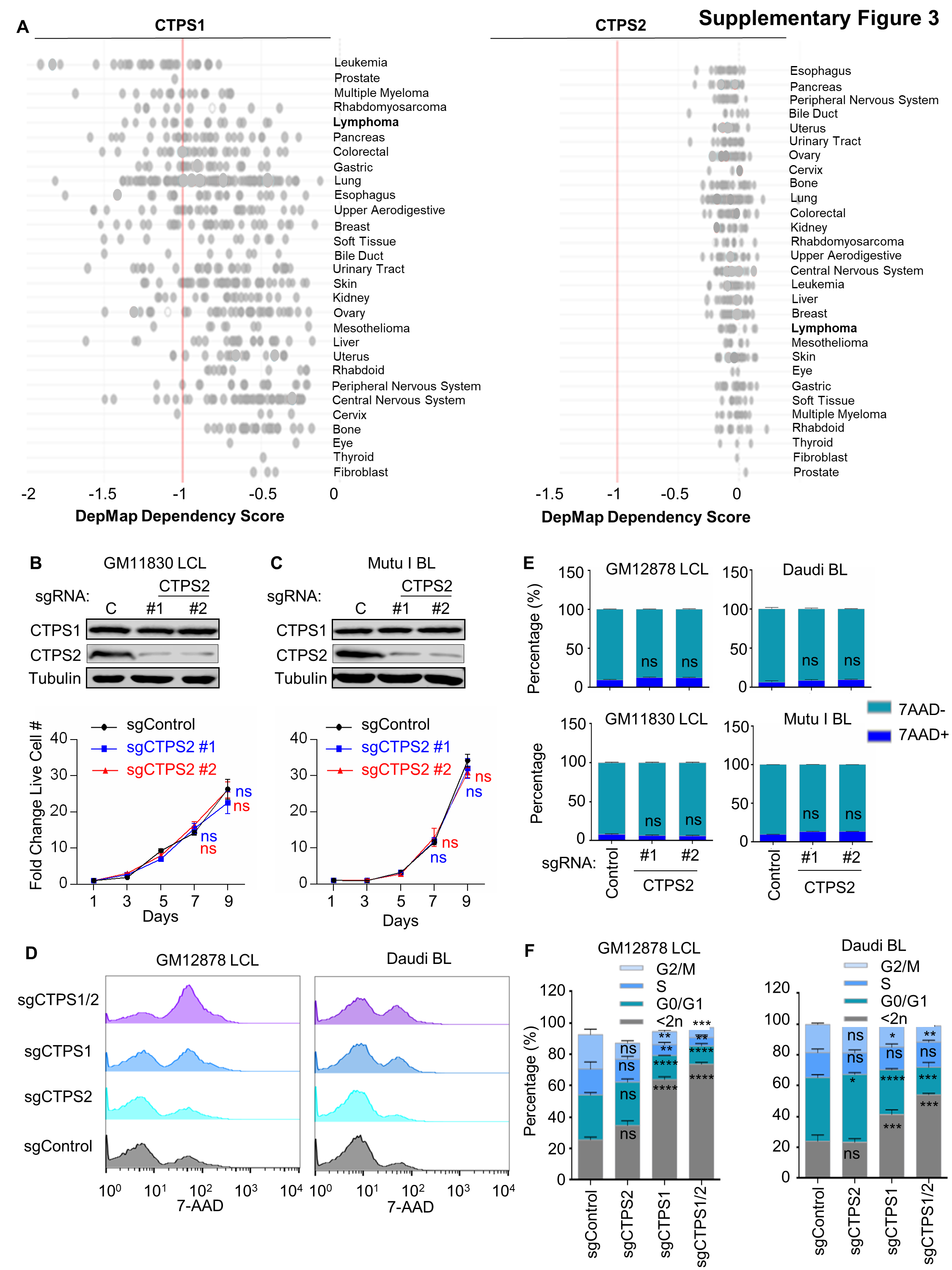

Supplement: FIG S3 [file mbio.01530-21-sf003.tif]

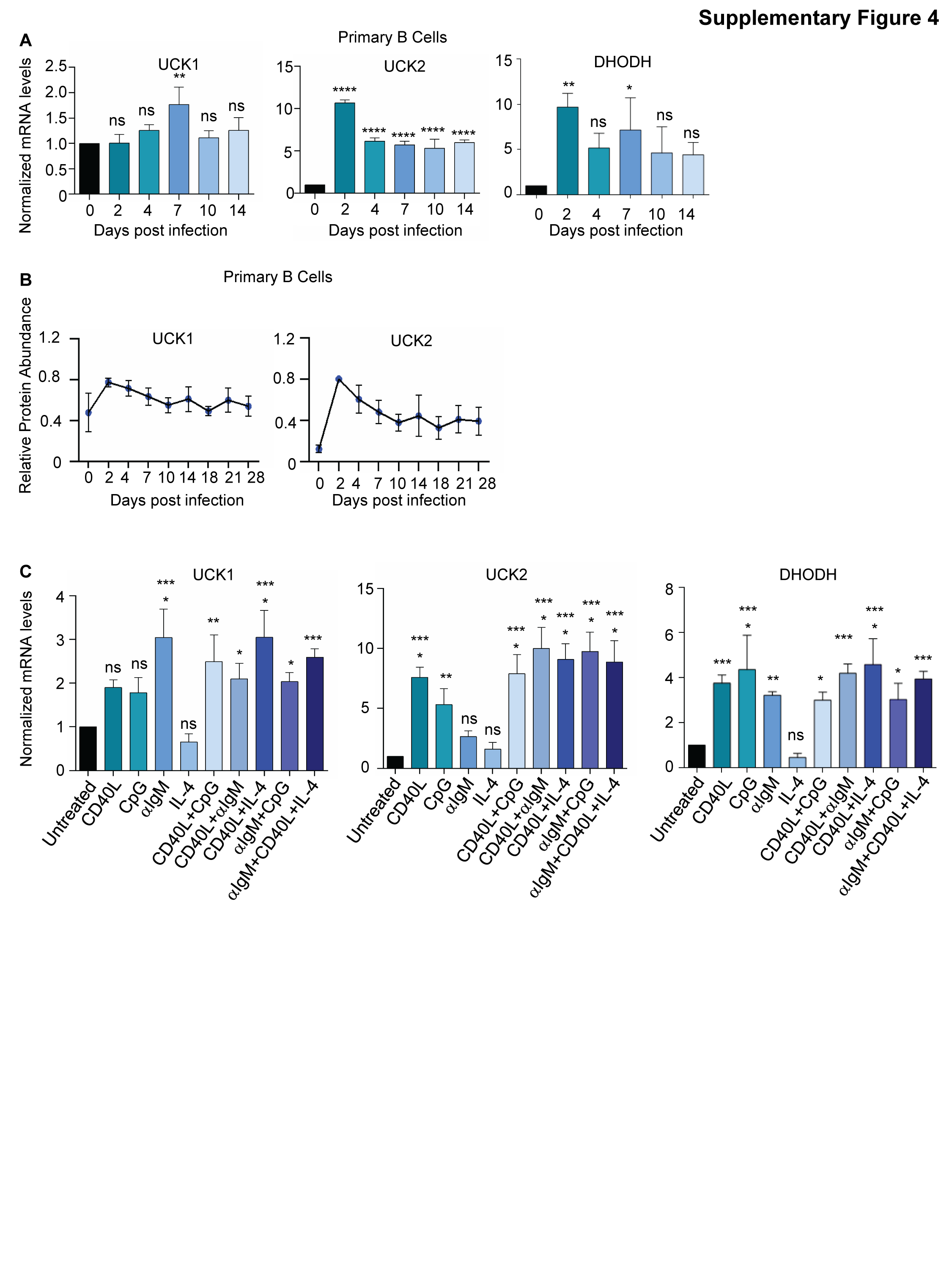

Supplement: FIG S4 [file mbio.01530-21-sf004.tif]

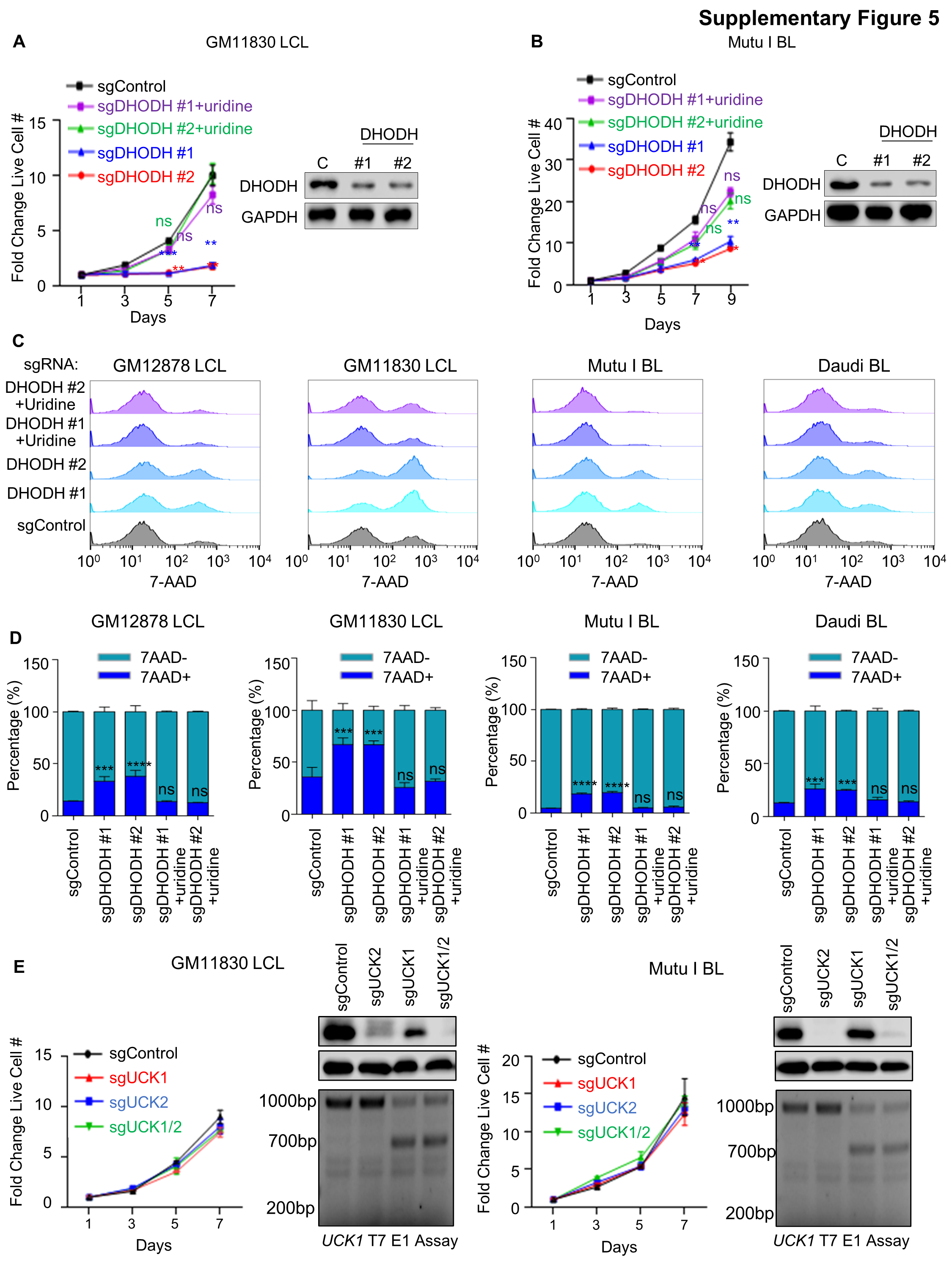

Supplement: FIG S5 [file mbio.01530-21-sf005.tif]

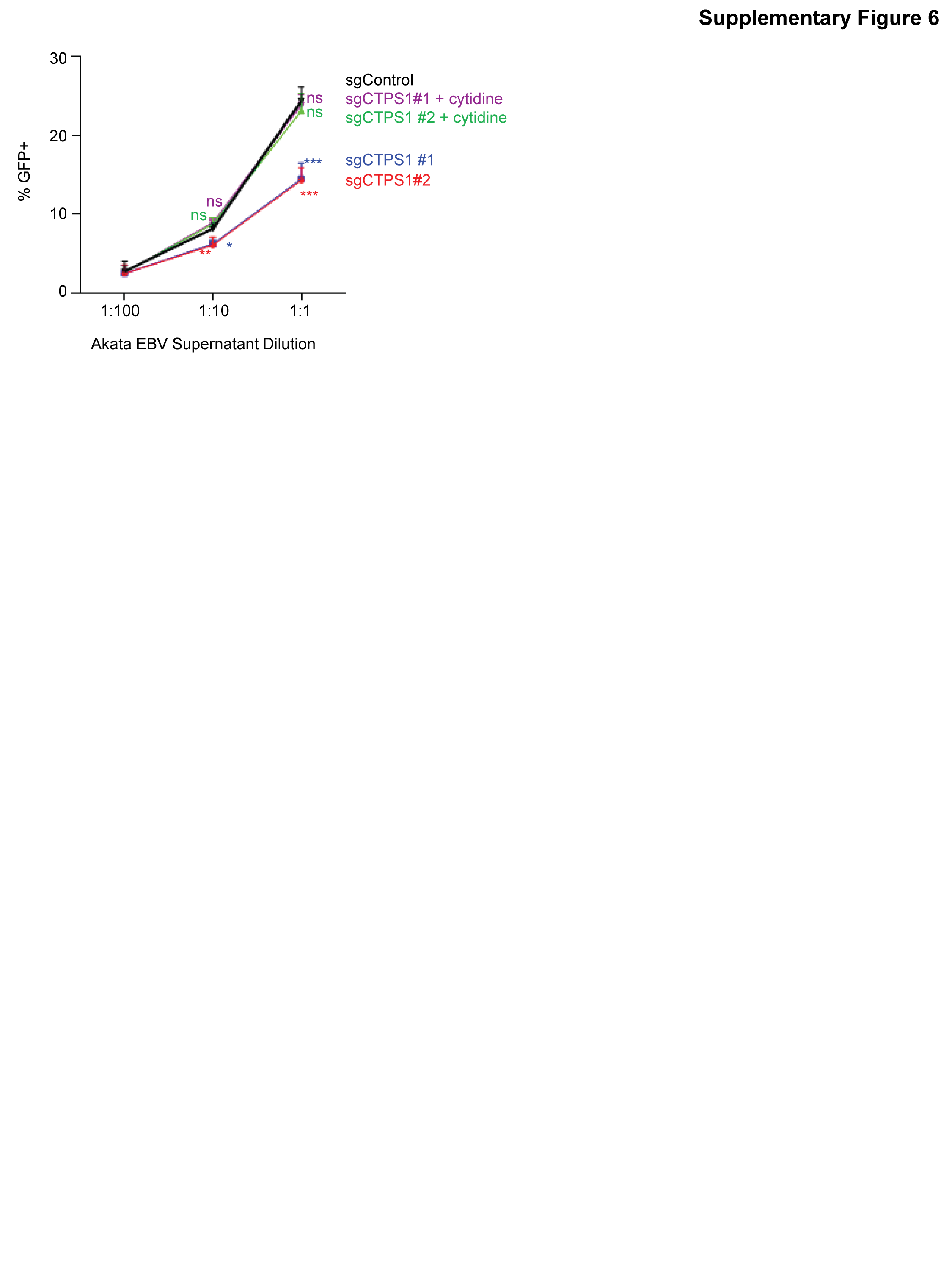

Supplement: FIG S6 [file mbio.01530-21-sf006.tif]
