# Supplementary material for: Epstein-Barr Virus Induced Cytidine Metabolism Roles in Transformed B-Cell Growth and Survival
Source: mBio. 2021 Jul 20;12(4):e01530-21. doi: 10.1128/mBio.01530-21 (PMC8406234; doi:10.1128/mBio.01530-21)
Supplement: TABLE S1 [file mbio.01530-21-st001.docx]

**Table S1** Sequences of sgRNAs used for CRISPR editing and CRISPRi

| sgRNA name | sgRNA sequence |
| --- | --- |
| sgControl | ATTTCGCAGATCATCGACAT |
| CTPS1#1 SS | CTGGAAAGATATACCAGTAT |
| CTPS1#2 SS | CTTGTTGTTAGAGGAGCAAG |
| CTPS2#1 SS | AAAACAGCGTCCGCGCACTG |
| CTPS2#2 SS | ATCGGTGATTCTGCAAGTAA |
| UCK1#1 SS | GGAGTTGCTGGGACAGAACG |
| UCK1#2 SS | GACAGAACGAGGTGGAACAG |
| UCK2#1 SS | CGTGTATGACTTTGTCTCCC |
| UCK2#2 SS | TTCCTTATAGGCGTCAGCGG |
| DHODH#1 SS | GGATGCTGTGATCATCCTGG |
| DHODH #2 AS | ATAGAAACGCTCATCTCCCG |
| RelA#1 SS | TGGTCCTGTGTAGCCATTGA |
| RelB#1 SS | GGTCTGGCGACGCGGCGACT |
| cRel#1 SS | ATTGGGTTCGAGACAACAGG |
| P50#1 SS | ATGACAGAGGCGTGTATAAG |
| P52#1 SS | TGGCCCCTACCTGGTGATCG |
| CTPS1 sg#1 (CRISPR-i) | CTGAAGCACAAAGTACAACG |
| CTPS1 sg#2 (CRISPR-i) | TGTAGTCTCAGCTAACTGGG |
| CTPS1 sg#3 (CRISPR-i) | TAAGGTACTAGCTACTCAGG |
| CTPS1 sg#4 (CRISPR-i) | CCATTTTACAGAAAAAACGG |
| CTPS1 sg#5 (CRISPR-i) | TTAATGAATGATGAGCCACT |
| CTPS1 sg#6 (CRISPR-i) | TTGGCCAAAACAAGTCACG |
| CTPS1 sg#7 (CRISPR-i) | CACACGCCTTCCAGTCGGG |
| CTPS1 sg#8 (CRISPR-i) | TGAAGTCCTTGTCCTAAGTG |
| CTPS1 sg#9 (CRISPR-i) | TTCTGTCACTTTGATCACCG |
| CTPS1 sg#10 (CRISPR-i) | AGAAACAAGGTGACCGATG |
| CTPS1 sg#11(CRISPR-i) | ACACCTATAGTGACAGGAG |
| CTPS1 sg#12 (CRISPR-i) | TTGTAGAAGTCGATGTGTG |
