# Supplementary material for: Epstein-Barr Virus Induced Cytidine Metabolism Roles in Transformed B-Cell Growth and Survival
Source: mBio. 2021 Jul 20;12(4):e01530-21. doi: 10.1128/mBio.01530-21 (PMC8406234; doi:10.1128/mBio.01530-21)
Supplement: TABLE S2 [file mbio.01530-21-st002.docx]

**Table S2** T7EI assay and RT-qPCR primers used in this study

| Oligo name | Oligo sequence |
| --- | --- |
| UCK1#1_T7EI_F | AGCCCCAAGTCCGTGCTAAC |
| UCK1#1_ T7EI_R | GTAGGCTGGGCCTCTGCTTC |
| CTPS1_F | GGTATATCTTTCCAGTGGTCAG |
| CTPS1_R | CTCTCCTTATGAGCATGGTG |
| CTPS2_F | ACGGGTGGGGTCATCTCAG |
| CTPS2_R | TTTTATGGCAGTAACTCGGAGTC |
| UCK1_F | AGTTGCTGGGACAGAACGAG |
| UCK1_R | CTGCCGTCAGGACCTTGTAG |
| UCK2_F | GCCCTTCCTTATAGGCGTCAG |
| UCK2_R | CTTCTGGCGATAGTCCACCTC |
| 18S_F | CGGCTACCACATCCAAGGAA |
| 18S_R | GCTGGAATTACCGCGGCT |
| GAPDH F | AATGAAGGGGTCATTGATGG |
| GAPDH R | AAGGTGAAGGTCGGAGTCAA |
