# Supplementary material for: Epstein-Barr Virus Induced Cytidine Metabolism Roles in Transformed B-Cell Growth and Survival
Source: mBio. 2021 Jul 20;12(4):e01530-21. doi: 10.1128/mBio.01530-21 (PMC8406234; doi:10.1128/mBio.01530-21)
Supplement: TEXT S1 [file mbio.01530-21-s0001.pdf]

Supplementary Methods

Recombinant EBV. EBV B95-8 virus was produced from B95-8 cells with conditional BZLF1 expression. The P3HR-1 EBV strain was produced from P3HR-1 cells with conditional BZLF1 and BRFL1 expression. UV irradiation of B95-8 virus supernatants was performed at a cumulative intensity of 3J per square centimeter on ice, to prevent heat-induced virus degradation. The quantitation of equal infection of B95-8, UV B 95-8 and P3HR1 was performed as previously described (29).

Flow cytometry analysis. 1 million live cells were washed twice with FACS buffer (PBS, 2%FBS), incubated with primary antibody for 30min on ice, washed three times with FACS buffer, and analyzed on a BD FACSCalibur instrument. FACS data were analyzed with FlowJo V10.

Cell cycle and cell death analysis

Cell cycle analysis was performed by staining fixed cells with propidium iodide (PI). Briefly, cells were pelleted by centrifugation at 300g, washed once and resuspended in 100µL PBS. 900uL ethanol was then added dropwise to fix cells and incubated at 4°C for 2 hours. Fixed cells were subsequently warmed for 5 minutes at room temperature, centrifuged at 300g, rehydrated with 1mL cold PBS for 5 minutes, centrifuged at 300g, and stained using 500µL staining solution (0.1% Triton X-100, 0.1mg/mL RNAse A (Thermo Fisher Cat#EN0531), 0.05mg/mL PI (Invitrogen Cat#P3566) in PBS) for 30 minutes before immediate processing by the flow cytometer . For 7-AAD staining, cells were stained with 1 µM 7-AAD (Fisher Scientific Cat#A1310) at room temperature for 5 minutes before being placed at ice until further processing with the flow cytometer.

Immunoblot analysis

Western blot analysis was performed as previously described (33). In brief, whole cell lysates were separated by SDS PAGE electrophoresis, transferred to nitrocellulose membranes, blocked with 5% milk in TBST and probed with primary antibodies at 4 degrees C overnight, followed by secondary antibody (Cell Signaling) for 1 h at room temperature.

Blots were then developed by incubation with ECL chemiluminescence for 1 min and images were captured by Li-Cor Fc platform. Immunoblot analysis was performed using the antibodies against the following targets at a 1:1000 dilution unless otherwise stated: anti-CTPS1 (Abcam Cat#Ab133743), anti-CTPS2 (Sigma Cat# HPA017437), anti-UCK2 (Proteintech Cat#14877-1-AP), anti-DHODH (Proteintech Cat#10511-1-AP), anti-DDX1 (Bethyl Cat#A300-521A-T), anti-

MYC (Cat# sc-764, Santa Cruz), anti-Tubulin (Abcam Cat# ab7291), anti-cleaved PARP (Cell Signaling Cat#5625p), anti-p-γH2AX (Millipore Cat#05-636), anti-RelA (Cell Signaling Cat# 8242), anti-RelB (Cell Signaling Cat# 4922), anti-cRel (Cell Signaling Cat# 4727S), anti-p50 antibody (Santa Cruz Cat# SC-1190X), anti-p52 (Millipore Sigma Cat#05-361), anti-p44/42 MAPK ERK 1/2 (Cell Signaling Cat#9102s), anti-pERK (T202/Y204) (Cell Signaling Cat#9101s), anti-EBNA2 (PE2) was obtained from Dr. Elliot Kieff, anti-BMRF1(OT14E2) was obtained from Dr. Jaap Middeldrop, anti-BZLF1 (Santa Cruz Cat# sc-53904), anti-GAPDH (Proteintech Cat#60004-1-Ig), anti-Mouse IgG, HRP-coupled secondary antibody (Cell Signaling Cat#7076), anti-Rabbit IgG, HRP-coupled secondary antibody (Cell Signaling Cat#7074).
